# Factors Associated With Benefit of Treatment of Patent Ductus Arteriosus in Preterm Infants: A Systematic Review and Meta-Analysis

**DOI:** 10.3389/fped.2021.626262

**Published:** 2021-02-09

**Authors:** Esther J. S. Jansen, Tim Hundscheid, Wes Onland, Elisabeth M. W. Kooi, Peter Andriessen, Willem P. de Boode

**Affiliations:** ^1^Radboud University Medical Center Nijmegen, Radboud Institute for Health Sciences, Amalia Children's Hospital, Nijmegen, Netherlands; ^2^Emma Children's Hospital, Amsterdam University Medical Centers, VU University Medical Center, University of Amsterdam, Amsterdam, Netherlands; ^3^University Medical Center Groningen, Beatrix Children's Hospital, University of Groningen, Groningen, Netherlands; ^4^Máxima Medical Center, Veldhoven, Netherlands; ^5^Department of Applied Physics, School of Medical Physics and Engineering, Eindhoven University of Technology, Eindhoven, Netherlands

**Keywords:** ductus botalli, patent ductus arteriosis, premature (babies), ibuprofen, indometacin, acetaminophen

## Abstract

**Context:** There is an ongoing debate on the optimal management of patent ductus arteriosus (PDA) in preterm infants. Identifying subgroup of infants who would benefit from pharmacological treatment might help.

**Objective:** To investigate the modulating effect of the differences in methodological quality, the rate of open-label treatment, and patient characteristics on relevant outcome measures in randomized controlled trials (RCTs).

**Data Sources:** Electronic database search between 1950 and May 2020.

**Study Selection:** RCTs that assessed pharmacological treatment compared to placebo/no treatment.

**Data Extraction:** Data is extracted following the PRISMA guidelines. Outcome measures were failure to ductal closure, surgical ligation, incidence of necrotizing enterocolitis, bronchopulmonary dysplasia, sepsis, periventricular leukomalacia, intraventricular hemorrhage (IVH) grade ≥3, retinopathy of prematurity and mortality.

**Results:** Forty-seven studies were eligible. The incidence of IVH grade ≥3 was lower in the treated infants compared to the placebo/no treatment (RR 0.77, 95% CI 0.64–0.94) and in the subgroups of infants with either a gestational age <28 weeks (RR 0.77, 95% CI 0.61–0.98), a birth weight <1,000 g (RR 0.77, 95% CI 0.61–0.97), or if untargeted treatment with indomethacin was started <24 h after birth (RR 0.70, 95% CI 0.54–0.90).

**Limitations:** Statistical heterogeneity caused by missing data and variable definitions of outcome parameters.

**Conclusions:** Although the quality of evidence is low, this meta-analysis suggests that pharmacological treatment of PDA reduces severe IVH in extremely preterm, extremely low birth weight infants or if treatment with indomethacin was started <24 h after birth. No other beneficial effects of pharmacological treatment were found.

## Introduction

Patent ductus arteriosus (PDA) is common in preterm and very low birth weight infants (1). Persistence is associated with a higher risk of morbidities, including bronchopulmonary dysplasia (BPD), necrotizing enterocolitis (NEC), and intraventricular hemorrhage (IVH), and mortality (2). Nevertheless, pharmacological treatment or surgical closure of PDA is not without adverse effects (3, 4). After many decades of clinical research, the question remains open if, when, and how PDA should be treated in preterm infants (5). Globally, there has been a shift from early pharmacological treatment toward a more expectant management policy (6). A uniform definition of a hemodynamic significant PDA does not exist, nor is there clear evidence in favor of or against many of the approaches to treating PDA (7–9). Since 1976 we know that pharmacological treatment is an effective way of ductal closure (10). A recent meta-analysis, however, showed that neither short-term nor long-term outcomes seem to differ between treated and untreated patients (11). This sparked an ongoing debate on the optimal approach to treating PDA, which ranges from expectant management to aggressive treatment with a variety of cyclooxygenase inhibitors or acetaminophen with varying doses and at different intervals (5). Although the results of randomized controlled trails (RCTs) on PDA treatment have been reviewed extensively, only a small number of reviews stratified the results according to infant characteristics, methodological quality (11, 12), timing of treatment (12), or to the definitions of a hemodynamic significant PDA (9).

To the best of our knowledge this is the first comprehensive systematic review of RCTs to investigate the modulating effect of the methodological quality, the rate of open-label treatment in the placebo/no treatment groups, and several patient characteristics on the benefits, or adverse effects, of pharmacological treatment of PDA in preterm infants. We aim to identify specific subgroups of preterm infants at high risk of adverse outcomes, who would benefit from active closure of PDA.

## Methods

Our study is performed in accordance with the Preferred Reporting Items for Systematic Reviews and Meta-Analyses (PRISMA) guidelines (13).

### Search Strategy

We searched the following databases: PubMed, the Ovid Embase, and the Cochrane Library. We searched for papers published between 1950 up to and including April 2020. By using the Boolean operators AND and OR, we used all possible combinations of the following search terms: infant, newborn, neonate, preterm, premature, ductus, arteriosus, Botalli. We also used the Mesh terms “Infant, premature,” “Ductus Arteriosus, Patent,” and “Ductus Arteriosus” in the PubMed database. The complete search strategy can be found in Supplement 1. Subsequently, we assessed the publications cited by the selected studies for relevant material eligible for possible additional inclusion.

### Study Selection

Three authors (EJ, TH, and WdB) independently screened the publications identified in our initial search for eligibility on the basis of their titles and abstracts. Where disagreement arose, the full text was assessed and then discussed in order to reach consensus. We selected studies with a RCT design and written in either English, Dutch, or German. Generally speaking, we included all studies that assessed pharmacological treatment with either ibuprofen, indomethacin, or acetaminophen vs. placebo/no treatment. We excluded animal studies, studies on antenatal treatment, studies that included patients with a post term age of more than 1 month, and studies concerning patients with a congenital heart defect.

### Data Extraction

Two authors (EJ and TH) performed data extraction. The data we extracted from the selected studies were general study parameters, demographic parameters pertaining to the participants, treatment regime(s), and outcomes. We collected the parameters study design, total number of patients, mean gestational age (GA), birth weight (BW), postnatal age (PNA) at the start of treatment, and the rate of open-label treatment in the placebo/no treatment group. The following outcome parameters were collected (if reported in the studies) and analyzed: mortality, failure to close the DA, the need for surgical ligation, the incidence of NEC (any definition), BPD (any definition), sepsis, periventricular leukomalacia (PVL), IVH grade ≥3, retinopathy of prematurity (ROP), oliguria, other respiratory morbidity (e.g., pneumothorax), other gastrointestinal morbidity [e.g., spontaneous intestinal perforation (SIP)], and long-term neurodevelopmental impairment. In case of missing data, we tried to contact the corresponding authors of the studies in question and requested them to kindly provide these data.

### Statistical Analysis

As ibuprofen, indomethacin, and acetaminophen are comparable regarding their effectiveness in DA closure (11, 14), but their side effect profiles may differ (11, 14), we performed two analyses. In the first analysis we combined all studies reporting either of these three drugs in comparison with placebo/no treatment. In a second analysis we divided the studies according to which drug was used. Subgroups were made, related to known risk factors (GA, BW) and other factors influencing efficacy of treatment, such as PNA. Moreover, the consequences of open label treatment percentage in the control group were analyzed since this is an important methodologic flaw in the RCTs. The following strata were analyzed: BW in five subgroups: <1,000 g, 1,000–1,250 g, 1,251–1,500 g, >1,500 g, and data unknown; GA in four subgroups: <28 weeks, 28–33 weeks, >33 weeks, and data unknown; PNA at the start of treatment in four subgroups: <24 h, 24–72 h, >72 h, and data unknown. Studies with start of treatment <24 h PNA were divided into untargeted (start treatment irrespective whether the ductus is open or closed) and targeted (start treatment only after clinically and/or echocardiographically confirmation of a PDA) treatment. The rate of open-label treatment in the placebo/no treatment arm was expressed as a percentage and divided into four groups: <25%, 25–50%, >50%, and data unknown. For statistical analysis we used Review Manager (RevMan version 5.3 Copenhagen: The Nordic Cochrane Center, The Cochrane Collaboration, 2014). The risk ratio (RR) and risk difference (RD) with a 95% confidence interval (CI) were calculated with the Mantel-Haenszel method. We calculated the number needed to treat (NNT) with a 95% CI for each different outcome in case of statistical significance. We used random-effect meta-analysis if the heterogeneity (*I*^2^) was >50% (15) and fixed-effect in case of low heterogeneity.

### Risk of Bias

We critically examined the methodological quality of the selected studies and the risk of bias in accordance with the Cochrane guidelines (16). The quality parameters included the type of analysis, random sequence generation, allocation concealment, blinding of participants and personnel, and blinding of outcome assessment. Two authors (EJ and TH) assessed the risk of bias assessment. When disagreement arose, a third author (WdB) assessed the studies in order to reach consensus. The risk of bias was calculated (low risk: 1 point, unclear risk: 2 points, and high risk: 3 points) and the cumulative score was divided into three subgroups: low (7–9 points in total), intermediate (10–12 points), and high (13–21 points). We examined the methodological quality of the studies' outcome parameters with the GRADE method (17). We assessed imprecision as serious if the total number of events was <300 or if the width of the CI of the RR was >0.25. We used the GRADE-pro GDT 2016 software [GRADEpro Guideline Development Tool (Software) McMaster University, 2015] to create a “summary of findings” table to report the quality of evidence. The GRADE approach results in an assessment of the quality of a body of evidence in one of four grades: high, moderate, low, or very low.

## Results

### Study Selection

**Figure 1:**
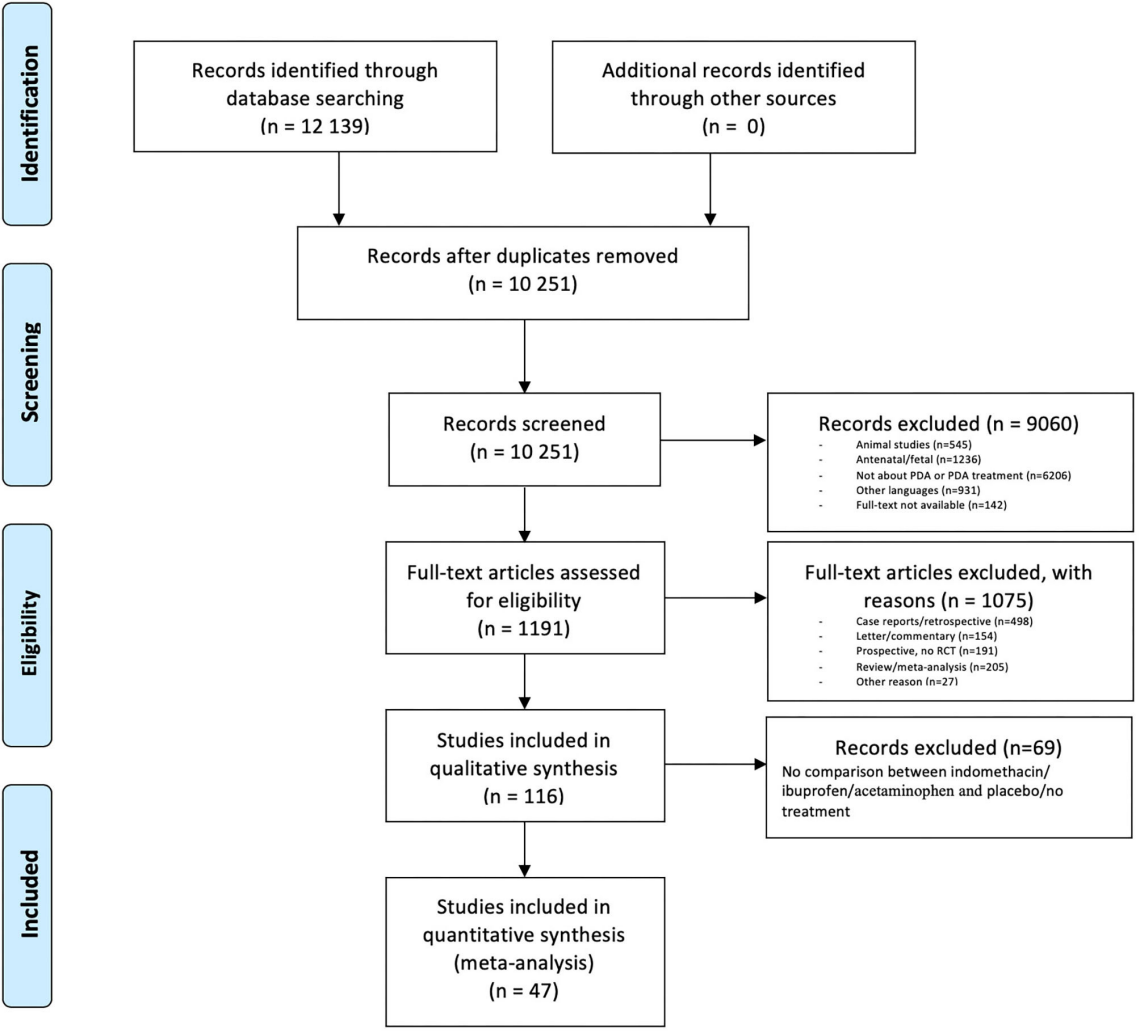
PRISMA flow diagram.

Out of the 12,139 articles we identified 10,251 as unique in our initial search. After selection (see Figure 1) a final 47 papers were eligible, comprising a total of 5,242 infants (18–64).

### Study Characteristics and Risk of Bias

The relevant characteristics of the 47 papers are described in Table 1 (18–64). Thirty-two (68%) of the studies analyzed the effect of indomethacin (18–44, 46, 47, 55, 60, 61), 12 (26%) studied the effect of ibuprofen (45, 48–54, 56–59), two (4%) studied acetaminophen (62, 64), and one (2%) studied the effect of either indomethacin, ibuprofen, or acetaminophen (63). Thirteen papers (28%) included preterm infants with a mean GA <28 weeks (42, 44, 46–48, 50, 53, 55–58, 61, 63), and 30 (64%) included infants with a mean GA between 28 and 32 weeks (18, 20–24, 26–30, 32–41, 43, 45, 47, 49, 51, 52, 59, 60, 62, 64). Seventeen papers (36%) included preterm infants with a mean BW <1,000 g (33, 35, 39, 41–46, 48, 50, 53, 55, 56, 58, 61, 63), and 19 (40%) included infants weighing between 1,000 and 1,250 g (20, 22–24, 27–30, 34, 36–38, 40, 47, 49, 52, 57, 62, 64). Most studies (62%) investigated treatment that was started within 24 h' PNA (26, 29, 30, 32, 34, 35, 37–53, 55, 58, 60–62, 64). More than two third of the studies reported the rate of open-label treatment, namely 25–50% in 16 (34%) (20, 22, 31, 32, 36, 37, 42, 46, 48, 50, 52, 53, 58, 60, 61, 63), and >50% in 15 (32%) studies (18, 19, 23–25, 27, 33, 34, 40, 43, 45, 47, 51, 55, 56). The median rate of the open-label treatment was 44.5% (range 0–85%). Twenty-one studies (45%) were classified as having a low risk of bias (18, 21–23, 25, 27, 29, 31, 33, 37, 38, 42, 43, 46, 47, 49, 50, 53, 56, 61, 62). Six papers (13%) were assessed as having a high risk of bias (19, 26, 34, 39, 40, 63).

**Table 1:** Treatment, patient and, study characteristics of the RCTs included.

**References**	**Patients (n)**	**Treatment characteristics**			**Patient characteristics**		**Study characteristics**								
		**Intervention**	**Control**	**Start at PNA**	**GA (w)**	**BW (g)**	**Open-label treatment**	**1**	**2**	**3**	**4**	**5**	**6**	**7**	**Risk of bias**
Nestrud et al. (18)	23	Indomethacin	Placebo	> 72 h	28–32	1,251–1,500	> 50%	+	+	+	?	+	+	?	Low
Merritt et al. (19)	24	Indomethacin	No treatment	24–72 h	unknown	1,251–1,500	> 50%	–	–	–	?	–	+	?	High
Neu et al. (20)	21	Indomethacin	Placebo	> 72 h	28–32	1,000–1,250	25–50%	?	+	+	+	–	+	?	Intermediate
Yanagi et al. (21)	17	Indomethacin	Placebo	> 72 h	28–32	1,251–1,500	unknown	+	+	+	?	+	+	?	Low
Yeh et al. (22)	55	Indomethacin	Placebo	> 72 h	28–32	1,000–1,250	25–50%	+	?	+	+	+	+	?	Low
Mahony et al. (23)	47	Indomethacin	Placebo	> 72 h	28–32	1,000–1,250	> 50%	+	+	+	+	+	+	?	Low
Mullett et al. (24)	47	Indomethacin	Placebo	> 72 h	28–32	1,000–1,250	> 50%	?	+	+	?	+	–	?	Intermediate
Gersony et al. (25)	405	Indomethacin	Placebo	> 72 h	unknown	unknown	> 50%	+	+	+	?	+	+	?	Low
Kaapa et al. (26)	27	Indomethacin	No treatment	<24 h T	28–32	>1,500	unknown	+	?	–	?	?	+	?	High
Rudd et al. (27)	30	Indomethacin	Placebo	> 72 h	28–32	1,000–1,250	> 50%	+	+	+	+	+	+	?	Low
Yeh et al. (28)	47	Indomethacin	Placebo	> 72 h	28–32	1,000–1,250	unknown	+	?	+	?	?	+	?	Intermediate
Mahony et al. (29)	104	Indomethacin	Placebo	<24 h UT	28–32	1,000–1,250	<25%	+	+	+	+	?	+	?	Low
Ment et al. (30)	48	Indomethacin	Placebo	<24 h UT	28–32	1,000–1,250	unknown	–	?	+	+	?	+	?	Intermediate
Hammerman et al. (31)	24	Indomethacin	Placebo	> 72 h	unknown	unknown	25–50%	+	+	+	+	?	+	?	Low
Rennie et al. (32)	50	Indomethacin	Placebo	<24 h UT	28–32	1,251–1,500	25–50%	?	?	+	+	?	?	?	Intermediate
Hammerman et al. (33)	24	Indomethacin	Placebo	> 72 h	28–32	<1,000	> 50%	+	+	+	+	+	+	?	Low
Krueger et al. (34)	32	Indomethacin	No treatment	<24 h UT	28–32	1,000–1,250	> 50%	?	?	–	?	?	+	?	High
Vincer et al. (35)	30	Indomethacin	Placebo	<24 h UT	28–32	<1,000	<25%	?	–	+	?	+	+	?	Intermediate
Weesner et al. (36)	26	Indomethacin	Placebo	24–72 h	28–32	1,000–1,250	25–50%	+	?	+	+	?	+	?	Intermediate
Bandstra et al. (37)	199	Indomethacin	Placebo	<24 h UT	28–32	1,000–1,250	25–50%	+	+	+	+	+	+	?	Low
Hanigan et al. (38)	111	Indomethacin	Placebo	<24 h UT	28–32	1,000–1,250	<25%	+	+	+	+	?	+	?	Low
Ment et al. (39)	36	Indomethacin	Placebo	<24 h UT	28–32	<1,000	unknown	–	?	+	+	–	+	?	High
Lai et al. (40)	32	Indomethacin	Placebo	<24 h UT	28–32	1,000–1,250	> 50%	–	–	?	?	?	+	?	High
Ment et al. (41)	431	Indomethacin	Placebo	<24 h UT	28–32	<1,000	<25%	+	+	?	+	–	+	?	Intermediate
Couser et al. (42)	90	Indomethacin	Placebo	<24 h UT	<28	<1,000	25–50%	+	?	+	+	+	+	?	Low
Supapannachart et al. (43)	30	Indomethacin	Placebo	<24 h UT	28–32	<1,000	> 50%	+	+	+	?	+	+	?	Low
Couser et al. (44)	90	Indomethacin	Placebo	<24 h UT	<28	<1,000	unknown	+	?	+	+	–	+	?	Intermediate
De Carolis et al. (45)	46	Ibuprofen	No treatment	<24 h UT	28–32	<1,000	> 50%	+	–	–	+	+	+	?	Intermediate
Schmidt et al. (46)	1,202	Indomethacin	Placebo	<24 h UT	<28	<1,000	25–50%	+	+	+	+	?	+	?	Low
Osborn et al. (47)	70	Indomethacin	Placebo	<24 h T	<28	1,000–1,250	> 50%	?	+	+	+	+	+	?	Low
Gournay et al. (48)	131	Ibuprofen	Placebo	<24 h UT	<28	<1,000	25–50%	+	+	+	+	–	+	–	Intermediate
van Overmeire et al. (49)	415	Ibuprofen	Placebo	<24 h UT	28–32	1,000–1,250	<25%	+	+	+	+	+	+	?	Low
Dani et al. (50)	155	Ibuprofen	Placebo	<24 h UT	<28	<1,000	25–50%	+	+	+	+	?	+	?	Low
Sangtawesin et al. (51)	42	Ibuprofen	Placebo	<24 h T	28–32	1,251–1,500	> 50%	?	?	+	+	+	+	?	Intermediate
Sangtawesin et al. (52)	62	Ibuprofen	Placebo	<24 h T	28–32	1,000–1,250	25–50%	?	?	+	+	+	+	?	Intermediate
Aranda et al. (53)	136	Ibuprofen	Placebo	24–72 h	<28	<1,000	25–50%	+	+	+	+	+	+	?	Low
Amoozgar et al. (54)	51	Ibuprofen	Placebo	> 72 h	> 33	>1,500	<25%	?	?	?	?	+	+	?	Intermediate
Maruyama et al. (55)	19	Indomethacin	Placebo	<24 h UT	<28	<1,000	> 50%	+	+	?	?	+	+	?	Intermediate
Sosenko et al. (56)	105	Ibuprofen	Placebo	24–72 h	<28	<1,000	> 50%	+	+	+	+	+	+	?	Low
Bagnoli et al. (57)	134	Ibuprofen	Placebo	> 72 h	<28	1,000–1,250	Unknown	?	?	?	?	+	+	?	Intermediate
Kanmaz et al. (58)	46	Ibuprofen	No treatment	<24 h UT	<28	<1,000	25–50%	+	+	–	+	–	+	?	Intermediate
Ding et al. (59)	72	Ibuprofen	Placebo	Unknown	28–32	1,251–1,500	Unknown	?	?	?	+	+	+	?	Intermediate
Jannatdoust et al. (60)	70	Indomethacin	No treatment	<24 h UT	28–32	Unknown	25–50%	+	+	–	?	?	+	?	Intermediate
Kluckow et al. (61)	92	Indomethacin	Placebo	<24 h T	<28	<1,000	25–50%	+	+	+	+	+	+	–	Low
Harkin et al. (62)	48	Acetaminophen	Placebo	<24 h UT	28–32	1,000–1,250	<25%	+	+	+	+	+	+	?	Low
Clyman et al. (63)	202	Any	No treatment	> 72 h	<28	<1,000	25–50%	+	?	–	?	+	+	–	High
Juujarvi et al. (64)	44	Acetaminophen	Placebo	<24 h UT	28–32	1,000–1,250	Unknown	+	+	+	+	?	?	?	Intermediate

*BW, birth weight; GA, gestational age; h, hour; n, number; NA, not available; PNA, postnatal age; any, indomethacin/ibuprofen/acetaminophen; T, targeted treatment, UT, untargeted treatment; risk of bias numbers stand for (1) random sequence generation (selection bias); (2) allocation concealment (selection bias); (3) blinding of participants and personnel (performance bias); (4) blinding of outcome assessment (detection bias); (5) incomplete outcome data (attrition bias); (6) selective reporting (reporting bias), and; (7) other bias*.

### Outcome Measures

Despite our efforts to contact the corresponding authors and our request to provide missing data, not all data on GA, BW and rate of open-label treatment could be retrieved. Data on GA (19, 25, 31) and/or BW (25, 31, 60) were unavailable in four trials (453 and 499 infants for GA and BW, respectively). The rate of open-label treatment was unavailable for nine trials (21, 26, 28, 30, 39, 44, 57, 59, 64).

RR of outcomes, stratified by the patient characteristics, the quality of the studies, and the rate of open-label treatment in the placebo/no treatment group are described in Table 2. The meta-analyses revealed that in comparison to placebo/no treatment, the administration of indomethacin, ibuprofen, or acetaminophen resulted in a significantly reduced risk of failed ductal closure (RR 0.40, 95% CI 0.33–0.48; RD −0.32, 95% CI −0.38, −0.27; NNT 3.4, 95% CI 3.1–3.7) or risk of surgical ligation (RR 0.61, 95% CI 0.49–0.76; RD −0.04, 95% CI −0.06, −0.02; NNT 22.8, 95% CI 15.8–40.7), irrespective of the used drug. This result was similar for the subcategories based on mean BW, GA, and PNA at the start of treatment. The quality of evidence was graded as very low or very low to low, respectively (Supplement 2).

**Table 2:** Risk ratio of outcomes, stratified by the patient characteristics, the quality of the studies, and the rate of open-label treatment in the placebo/no treatment group.

	**Failed closure PDA**	**Need for ligation**	**NEC**	**BPD**	**Sepsis**	**PVL**	**IVH (Grade ≥ 3)**	**ROP**	**Mortality**
Whole population	**0.40 [0.33–0.48]** **40; 4,291**	**0.61 [0.49–0.76]** **24; 3,289**	1.10 [0.89–1.37] 29; 3,686	0.97 [0.85–1.10] 26; 3,005	1.02 [0.82–1.25] 13; 1,205	0.93 [0.65–1.33] 11; 1,619	**0.76 [0.62–0.93]** **19; 3,071**	0.99 [0.86–1.15] 19; 1,489	1.02 [0.89–1.17] 37; 3,987
BW <1,000 g	**0.48 [0.37–0.63]** **13; 2,045**	**0.61 [0.46–0.80]** **11; 2,102**	1.07 [0.84–1.37] 14; 2,330	0.94 [0.79–1.12] 13; 1,983	1.09 [0.84–1.41] 7; 720	0.84 [0.55–1.28] 8; 951	**0.77 [0.61–0.97]** **12; 2,251**	1.18 [0.95–1.47] 9; 864	1.08 [0.91–1.28] 15; 2,368
BW 1,000–1,250 g	**0.37 [0.27–0.51]** **18; 1,516**	**0.60 [0.41–0.89]** **10; 1,088**	1.21 [0.74–1.97] 13; 1,291	1.07 [0.90–1.26] 8; 857	0.89 [0.61–1.29] 6; 485	1.22 [0.60–2.48] 3; 668	0.78 [0.54–1.13] 6; 851	0.82 [0.67–1.00] 9; 585	0.98 [0.75–1.28] 15; 1,413
BW 1,251–1,500 g	**0.32 [0.16–0.65]** **5; 204**	1.08 [0.08–15.46] 2; 75	1.27 [0.54–3.01] 1; 41	0.67 [0.10–4.53] 3; 114	-	-	-	1.36 [0.25–7.27] 1; 40	0.65 [0.33–1.28] 5; 155
BW > 1,500 g	0.15 [0.02–1.09] 1;27	-	-	0.54 [0.06–5.26] 1; 27	-	-	-	-	0.27 [0.03–2.11] 1; 27
BW unknown	**0.28 [0.18–0.44]** **3; 499**	0.56 [0.13–2.33] 1; 24	0.70 [0.07–6.70] 1; 24	1.05 [0.78–1.41] 1; 24	-	-	0.69 [0.12–3.85] 1; 69	-	1.40 [0.55–3.57] 1; 24
GA <28 w	**0.63 [0.47–0.84]** **9; 2,039**	**0.67 [0.52–0.87]** **9; 2,130**	1.20 [0.94–1.54] 12; 2,367	0.92 [0.74–1.15] 9; 1,858	1.15 [0.89–1.49] 5; 644	0.90 [0.59–1.37] 8; 959	**0.77 [0.61–0.98]** **9; 2,008**	1.17 [0.94–1.46] 7; 809	1.05 [0.88–1.25] 12; 2,369
GA 28–32 w	**0.34 [0.28–0.41]** **29; 1,823**	**0.45 [0.29–0.71]** **13; 1,110**	0.86 [0.56–1.34] 16; 1,295	1.06 [0.92–1.21] 15; 1,100	0.83 [0.58–1.18] 8; 561	1.02 [0.50–2.04] 3; 660	0.77 [0.55–1.09] 10; 1,063	0.86 [0.71–1.04] 12; 680	0.97 [0.76–1.24] 23; 1,570
GA > 33 w	-	-	-	-	-	-	-	-	-
GA unknown	**0.31 [0.22–0.42]** **2; 429**	0.66 [0.19–2.28] 2; 49	0.70 [0.07–6.70] 1; 24	0.57 [0.08–4.09] 2; 47	-	-	-	-	0.88 [0.38–2.04] 2; 48
PNA <24 h (targeted)	0.59 [0.25–1.35] 3; 159	0.22 [0.01–4.41] 1; 92	1.56 [0.45–5.42] 3; 224	0.68 [0.42–1.09] 3; 177	0.85 [0.48–1.49] 1; 92	1.70 [0.54–5.37] 2; 146	-	0.46 [0.16–1.32] 2; 150	0.76 [0.41–1.42] 3; 189
PNA <24 h (untargeted)	**0.39 [0.32–0.48]** **21; 2,916**	**0.53 [0.39–0.72]** **13; 2,502**	1.04 [0.79–1.36] 17; 2,677	0.96 [0.75–1.22] 14; 2,235	0.95 [0.69–1.32] 8; 704	0.82 [0.50–1.33] 6; 1,036	**0.70 [0.57–0.87]** **16; 2,630**	1.02 [0.85–1.21] 9; 706	1.04 [0.88–1.21] 20; 2,872
PNA 24–72 h	**0.12 [0.02–0.63]** **2; 68**	0.78 [0.35–1.76] 2; 130	1.26 [0.71–2.22] 3; 276	0.93 [0.61–1.43] 5; 288	-	0.58 [0.18–1.83] 2; 235	0.92 [0.50–1.70] 2;239	1.20 [0.90–1.61] 4; 294	0.77 [0.42–1.42] 5; 289
PNA > 72 h	**0.41 [0.30–0.57]** **13;1,076**	0.75 [0.52–1.07] 8; 565	1.33 [0.87–2.04] 7; 907	[0.86–1.19] 4; 305	1.15 [0.84–1.58] 4; 409	1.20 [0.57–2.51] 1; 202	1.63 [0.82–3.24] 1; 202	0.78 [0.48–1.27] 4; 339	1.21 [0.81–1.81] 9; 637
PNA unknown	0.13 [0.02–1.00] 1; 72	-	-	-	-	-	-	-	-
Low risk of bias	**0.39 [0.30–0.50]** **18; 3,049**	**0.55 [0.42–0.71]** **13; 2,548**	1.04 [0.80–1.35] 15; 2,767	0.97 [0.83–1.14] 13; 2,284	0.92 [0.73–1.18] 10; 925	0.85 [0.53–1.36] 8; 1,240	**0.79 [0.63–0.98]** **9; 2,393**	[0.86–1.17] 11; 1,029	1.03 [0.88–1.21] 19; 2,940
Intermediate risk of bias	**0.37 [0.26–0.53]** **17; 927**	0.74 [0.46–1.20] 8; 483	1.61 [0.98–2.64] 11; 655	1.03 [0.74–1.44] 8; 408	[0.07–15.04] 1; 46	0.90 [0.36–2.22] 2; 177	**0.55 [0.32–0.96]** **7; 410**	0.83 [0.48–1.42] 6; 226	0.98 [0.70–1.38] 12; 694
High risk of bias	**0.42 [0.19–0.97]** **5; 315**	0.90 [0.45–1.82] 3; 258	0.84 [0.47–1.53] 3; 264	0.88 [0.68–1.13] 5; 313	1.40 [0.89–2.21] 2; 234	1.20 [0.57–2.51] 1; 202	1.02 [0.57–1.82] 3; 268	1.12 [0.68–1.86] 2; 234	1.05 [0.62–1.77] 6; 353
Open–label treatment <25%	**0.38 [0.29–0.50]** **6; 762**	**0.39 [0.15–0.97]** **3; 549**	0.65 [0.31–1.33] 5; 658	1.09 [0.90–1.31] 3; 493	0.97 [0.47–1.99] 2; 152	2.05 [0.71–5.89] 1; 415	1.06 [0.63–1.78] 4; 635	0.93 [0.45–1.93] 3; 164	1.14 [0.81–1.62] 6; 769
Open–label treatment 25–50%	**0.40 [0.31–0.52]** **14; 2,323**	**0.52 [0.39–0.70]** **11; 2,226**	1.09 [0.85–1.39] 12; 2,379	0.94 [0.78–1.13] 14; 2,160	1.07 [0.82–1.39] 6; 793	0.72 [0.47–1.11] 7; 999	**0.76 [0.61–0.95]** **9; 2,152**	[0.87–1.17] 10; 1,036	1.05 [0.89–1.24] 14; 2,457
Open–label treatment > 50%	**0.40 [0.25–0.62]** **13; 843**	**0.63 [0.40–0.99]** **8; 361**	1.24 [0.68–2.28] 10; 467	0.98 [0.76–1.26] 8; 325	0.90 [0.61–1.34] 5; 260	1.63 [0.58–4.53] 3; 205	0.57 [0.25–1.30] 4; 200	1.17 [0.64–2.15] 4; 212	0.86 [0.55–1.33] 12; 499
Open–label treatment unknown	**0.37 [0.21–0.66]** **7; 363**	1.89 [0.91–3.93] 1; 134	[0.07–15.08] 1; 48	0.54 [0.06–5.26] 1; 27	-	-	0.26 [0.05–1.48] 2; 84	0.32 [0.07–1.42] 1; 47	0.41 [0.15-1.13] 5; 262

*Risk ratio with 95% confidence interval. Number of studies and infants. RR <1 favors treatment; RR > 1 favors placebo/no treatment. In bold the statistically significant differences. BW, birth weight; GA, gestational age; PNA, postnatal age; PDA, patent ductus arteriosus; NEC, necrotizing enterocolitis; BPD, bronchopulmonary dysplasia; PVL, periventricular leukomalacia; IVH, intraventricular hemorrhage; ROP, retinopathy of prematurity. w, weeks; h, hours. - means not estimable*.

We found no difference for BPD, NEC, sepsis, PVL, ROP or mortality between the intervention and control group overall, or in any of the subgroups (Table 2), irrespective of the used drug. In most studies BPD was defined as supplemental oxygen requirement at 28 days' PNA or at 36 weeks' postmenstrual age (PMA). Seven RCTs used radiographic criteria (19, 22, 26, 31, 33, 35, 36). Four RCTs did not state their definition of BPD clearly (32, 43, 51, 52). Neither the overall meta-analyses nor the subgroup analyses of the 28 days' PNA and 36 weeks' PMA definition of BPD revealed any differences between the placebo/no treatment and the pharmacological treatment group.

Twenty-eight out of 47 studies started the treatment <24 h PNA. Of these 28 studies, five started treatment only after clinically and/or echocardiographically confirmation of a PDA (targeted treatment) (26, 47, 51, 52, 61). All the other RCTs started irrespective whether the ductus was open or closed within the first 24 h after birth (untargeted treatment).

Compared to the no treatment group, the infants allocated to the pharmacological treatment group had a lower risk of IVH grade ≥3 (RR 0.76, 95% CI 0.62–0.93; RD −0.03, 95% CI −0.05, −0.01; NNT 34, 95% CI 18.9–136.6). This reduced risk of IVH grade ≥3 was also observed in the subgroups GA <28 weeks (RR 0.77, 95% CI 0.61–0.98; RD −0.03, 95% CI −0.06, −0.00; NNT 30.3, 95% CI 16.1–262.9), BW <1,000 g (RR 0.77, 95% CI 0.61–0.97; RD −0.03, 95% CI −0.06, −0.00; NNT 30.2, 95% CI 16.4–199.9), or if treatment was given untargeted <24 h' PNA (RR 0.70; 95% CI 0.57–0.87; RD −0.04, 95% CI −0.06, −0.02; NNT 26, 95% CI 15.7–64.1).

**Figure 2:**
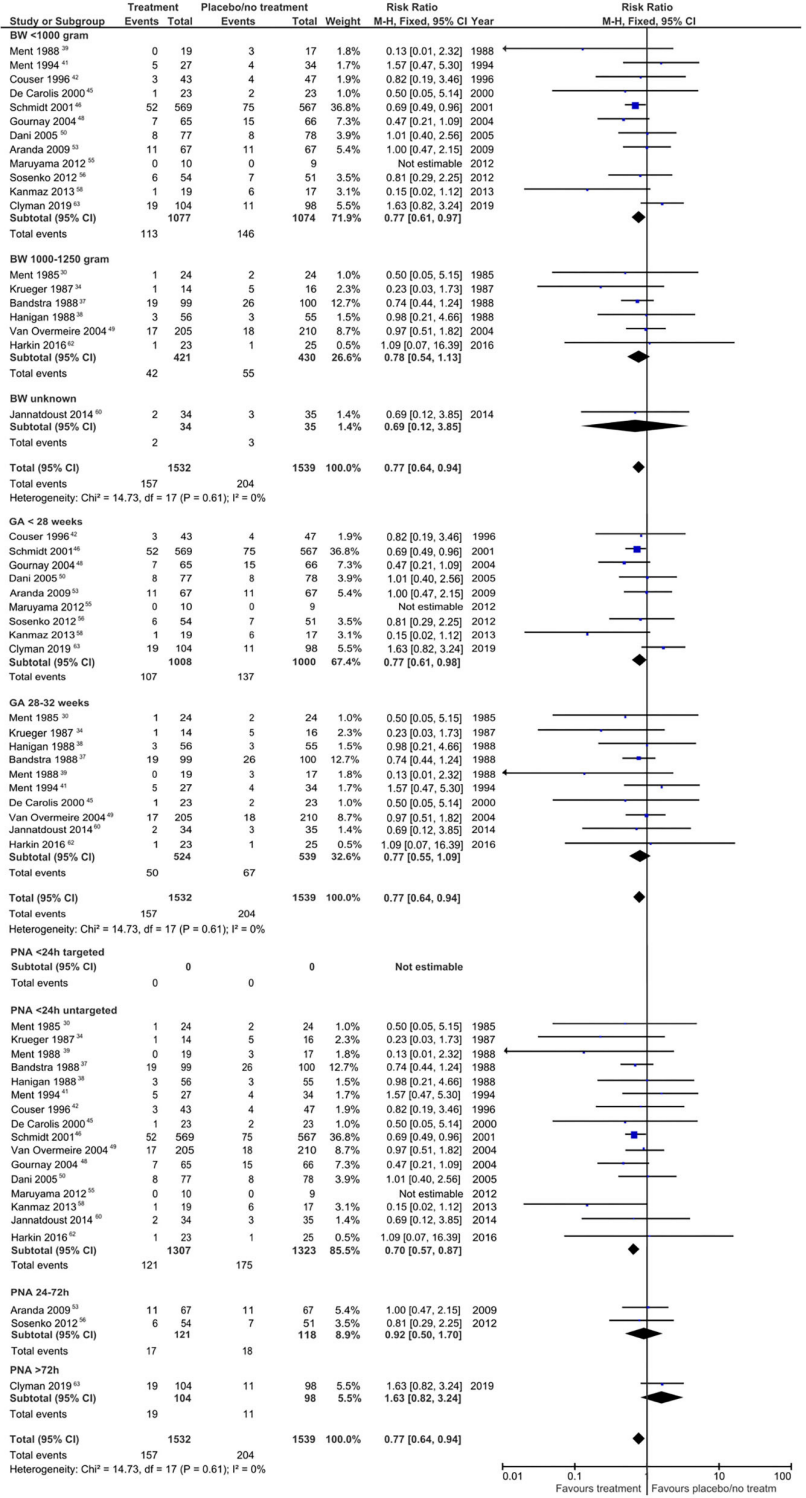
Forest plots regarding the risk for intraventricular hemorrhage grade ≥ 3. BW, birth weight; GA, gestational age; PNA, postnatal age.

We found a significant reduction in severe IVH only when untargeted treatment with indomethacin was used <24 h PNA compared to no treatment (RR 0.70, 95% CI 0.54–0.90; RD −0.04, 95% CI −0.07, −0.01). Forest plots for the risk of IVH grade ≥3 are depicted for the different subgroups in Figure 2. Furthermore, the incidence of IVH grade ≥3 in the treatment group was significantly lower in the low and intermediate risk of bias groups and if the rate of open-label treatment was 25–50%. The quality of evidence was graded as very low to low (Supplement 2).

Subgroup analyses of the other outcome measurements proved impossible on account of the scarcity of available data. Five RCTs described data on oliguria (37, 40–42, 62). Five studies described the incidence of pneumothorax (30, 32, 37–39), six studies pulmonary hemorrhage (37, 42, 46, 60, 61, 63), and one study reported pulmonary hypertension as outcome measure (60). Five studies described the incidence of gastrointestinal bleeding (24, 27, 32, 40, 61), while two studies reported the incidence of SIP (46, 61). Only three RCTs described the long-term data on neurodevelopmental outcomes regarding motor delay, cognitive delay, the incidence of deafness and blindness, and neurodevelopmental impairment in general (44, 46, 64).

## Discussion

### Summary of Evidence

The aim of this systematic review was to investigate whether patient characteristics or study characteristics modulate the beneficial or adverse effects of PDA treatment in preterm infants. The main finding of this review was that pharmacologic treatment of PDA is associated with a significantly reduced risk of IVH grade ≥3 in extremely preterm infants (GA <28 weeks), extremely low BW infants (BW <1,000 g), or when untargeted treatment with indomethacin was started <24 h after birth. Moreover, this review revealed no relevant significant differences for the outcome measures NEC, BPD, mortality, sepsis, PVL, and ROP between intervention and control groups in the subgroups BW, GA, risk of bias, and rate of open-label treatment.

**Figure 3:**
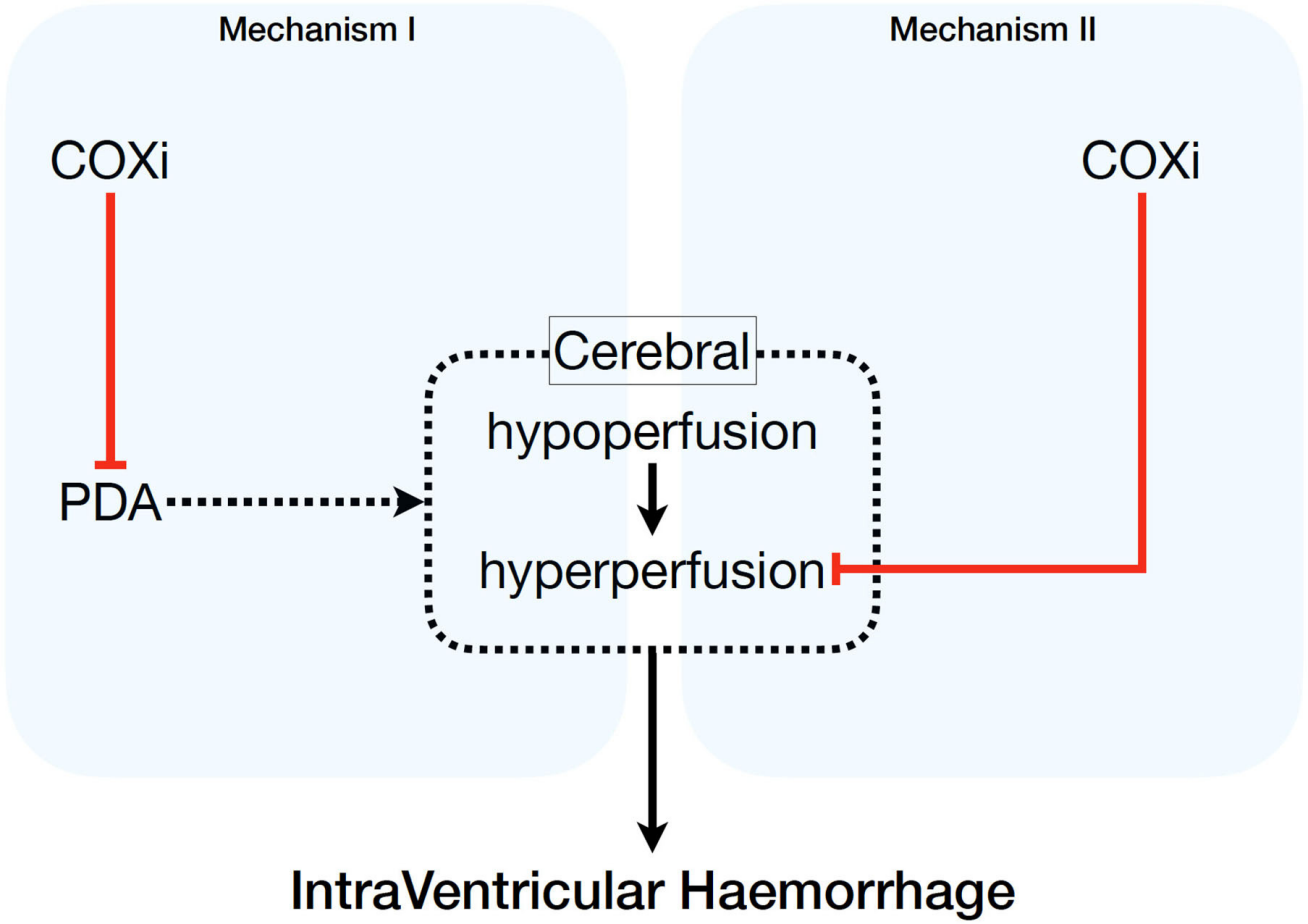
Potential pathophysiological mechanisms of pharmacologic cyclo-oxygenase inhibition on intraventricular hemorrhage. COXi, cyclo-oxygenase; PDA, patent ductus arteriosus.

Our findings regarding the reduced risk of IVH grade ≥3 is in line with a previous review comprising 2,588 newborns <37 weeks' gestation, which showed that untargeted administration of indomethacin is associated with a decreased risk of IVH (65). In our meta-analysis a total of 2,937 preterm infants were assessed for IVH grade ≥3 and stratified by BW, GA, and PNA. Out of the infants allocated to the treatment group 10% had IVH grade ≥3 compared to 13% of the infants in the placebo/no treatment group. Dividing the included studies who treated the infants <24 h after birth into untargeted treatment or targeted treatment, we found only reduction of severe IVH in the former group if indomethacin was used. The hypothesis is that this reduction of severe IVH is probably not a direct effect of ductal closure itself and therefore limiting cerebral perfusion disturbances, but mediated by prevention of hyperperfusion by a direct drug-induced cerebrovascular vasoconstriction (see Figure 3). This effect has been demonstrated for indomethacin and might prevent the cerebral hypoperfusion-hyperperfusion sequence, which is considered to be an important pathophysiological mechanism associated with IVH (66–69).

Subdividing the studies according to which drug was used, we found no significant differences in the incidences of NEC, BPD, ROP or mortality, which is in line with recently published papers (11, 14). In contrast to our meta-analysis, these papers used any grade of IVH instead of severe IVH as outcome parameter and observed no significant differences in any of the used drugs vs. placebo/no treatment. Our review selected studies published between 1985 and 2019, whereas currently, as opposed to the previous century, most preterm infants will have received corticosteroids antenatally and surfactants postnatally, if required. We know that this approach reduces the risk of an IVH (70). Including only those studies published in the last 25 years, the significant reduction of severe IVH is still observed in the youngest, smallest and untargeted treated infants.

Although untargeted treatment constitutes the only convincing evidence for active closure of PDA, it is currently seldom provided (1). It might, however, be argued that any evidence-based reduction in the risk of IVH grade ≥3 is beneficial to the infant, but sufficient evidence is lacking. In a 2015 meta-analysis about neurodevelopmental impairment after a severe IVH, only observational cohort studies were identified and on the whole the risk of bias was high (71). Moreover, there is also little evidence of improved long-term developmental outcome and mortality after prophylactic treatment (44, 45, 65).

In addition, we stratified by rate of open-label treatment, something that to our knowledge has not been done before. The median rate of the open-label treatment in the studies was 44.5% (calculated from 38 out of 47 studies). Since we reviewed the raw data in our meta-analysis to determine the morbidity and mortality of the different subgroups, we could not analyze whether the original studies performed intention to treat or per protocol analysis. We hypothesized that the potential effects of active treatment of a PDA would be attenuated in RCTs with a high proportion of open label treatment in the control/placebo arm. However, this was not observed. Failure of DA closure and the need for surgical ligation were significantly lower in the treatment group independent of the rate of open-label treatment. To our surprise, however, this subgroup analysis, which stratified the studies according to a high rate vs. a lower rate of open-label treatment in the control group, showed no difference in morbidity and mortality. We found no significant reduction of major clinical outcomes, not even in the subgroup of RCTs with low open-label treatment rates in the no treatment group of patients. This raises the question whether a PDA should be considered as an epiphenomenon as was suggested by recent cohort studies using restrictive treatment policies (72, 73). This should, however, be supported or refuted by well-powered high-quality RCTs targeting the high-risk population (<28 weeks' GA and/or BW <1,000 g) with low-rate open-label treatment of the placebo/no treatment group.

### Limitations

The first limitation of this meta-analysis are the missing data in the RCTs. Unfortunately, even though we tried to reduce selection bias by contacting the corresponding authors, not all missing data could be retrieved. As a consequence, we could not include all studies in our subgroup stratification.

Secondly, a meta-analysis has to deal with heterogeneity of the included RCTs. The high statistical heterogeneity in this meta-analysis is comparable with a previously published meta-analyses (74). Heterogeneity leads to lower quality of evidence (5, 75). In an attempt to reduce clinical heterogeneity, we stratified the results in several subgroups and subdivided treatment started <24 h after birth in untargeted treatment and targeted treatment. Moreover, the definitions of outcome measures in the included RCTs in a meta-analysis vary. In the current meta-analysis, the outcomes BPD and NEC were not uniformly defined in the selected studies. The possible reason is the large spread in publication years; criteria for short-term morbidities have changed over the years. Nevertheless, neither the overall meta-analyses nor the subgroup analyses of the different BPD definitions revealed any differences. The heterogeneity of studies analyzing NEC was low. Unfortunately, subgroup analyses could not be performed for the outcome measures pneumothorax, pulmonary hemorrhage, pulmonary hypertension, gastro-intestinal bleeding, SIP, and oliguria, because of the scarcity of available data. Last, another important factor that could be a major factor contributing to heterogeneity is the classification of hemodynamic significance of the PDA. Zonnenberg et al. showed that there is substantial variability in the definition of a significant PDA in clinical trials (9). In the 47 included RCTs the used definition of a PDA varied much, ranging from clinical, radiographic and echocardiographic parameters.

Future research is required with unambiguously definitions of outcome measures and larger groups of preterm infants. There is a need for well-powered high-quality RCTs with low-rate open-label treatment of the placebo/no treatment group. In addition, more research is needed to investigate which mechanisms might be responsible for the reduction of IVH grade ≥3 in the youngest, the smallest, or in the preterm infants that are treated untargeted with indomethacin within the first 24 h of life.

### Conclusions

In this systematic review, in which we investigated the modulating effects of patient characteristics and study characteristics by performing subgroup meta-analyses, the degree of heterogeneity among the included studies and variability in study quality is high. Therefore, the quality of evidence following GRADE assessment is low. Pharmacological treatment of a PDA in extremely preterm infants with either a GA <28 weeks, a BW <1,000 g, or if untargeted treatment with indomethacin is given <24 h PNA is associated with a significantly lower risk of developing IVH grade ≥3. We found no differences in the incidence of other morbidities or in mortality when we stratified the subgroups by BW, GA, and PNA at start of treatment. Important data on long-term consequences of neurodevelopmental impairment are lacking for these studies. More high-quality and low-rate open-label treatment studies are needed to unravel the effects of pharmacological PDA treatment on short-term and long-term morbidity and to elucidate underlying pathophysiologic mechanisms.

## Data Availability Statement

The raw data supporting the conclusions of this article will be made available by the authors, without undue reservation.

## Author Contributions

EJ conceptualized and designed the study, collected the data, drafted the initial manuscript, and analyzed and interpreted the data. TH collected, analyzed and interpreted the data, and reviewed and revised the manuscript. WO, EK, and PA analyzed and interpreted the data, critically reviewed the manuscript, and provided administrative, technical, or material support. WB coordinated and supervised data collection, analyzed data, and critically reviewed the manuscript for important intellectual content. All authors approved the final manuscript as submitted and agree to be accountable for all aspects of the work.

## Conflict of Interest

The authors declare that the research was conducted in the absence of any commercial or financial relationships that could be construed as a potential conflict of interest.

## References

[1] Lee JA Kim MJ Oh S Choi BM. Current status of therapeutic strategies for patent ductus arteriosus in very-low-birth-weight infants in Korea. J Korean Med Sci. (2015) 30(Suppl. 1):S59–66. 10.3346/jkms.2015.30.S1.S59 26566359 PMC4641065

[2] Sellmer A Bjerre JV Schmidt MR McNamara PJ Hjortdal VE Host B. Morbidity and mortality in preterm neonates with patent ductus arteriosus on day 3. Arch Dis Child Fetal Neonatal Ed. (2013) 98:F505–10. 10.1136/archdischild-2013-303816 23893268

[3] Heuchan AM Clyman RI. Managing the patent ductus arteriosus: current treatment options. Arch Dis Child Fetal Neonatal Ed. (2014) 99:F431–6. 10.1136/archdischild-2014-306176 24903455

[4] Bourgoin L Cipierre C Hauet Q Basset H Gournay V Roze JC. Neurodevelopmental outcome at 2 years of age according to patent ductus arteriosus management in very preterm infants. Neonatology. (2016) 109:139–46. 10.1159/000442278 26726863

[5] Sankar MN Bhombal S Benitz WE. PDA: to treat or not to treat. Congenit Heart Dis. (2019) 14:46–51. 10.1111/chd.12708 30811796

[6] Ngo S Profit J Gould JB Lee HC. Trends in patent ductus arteriosus diagnosis and management for very low birth weight infants. Pediatrics. (2017) 139:e20162390. 10.1542/peds.2016-2390 28562302 PMC5369670

[7] Bose CL Laughon MM. Patent ductus arteriosus: lack of evidence for common treatments. Arch Dis Child Fetal Neonatal Ed. (2007) 92:F498–502. 10.1136/adc.2005.092734 17951552 PMC2675405

[8] Clyman RI Couto J Murphy GM. Patent ductus arteriosus: are current neonatal treatment options better or worse than no treatment at all? Semin Perinatol. (2012) 36:123–9. 10.1053/j.semperi.2011.09.022 22414883 PMC3305915

[9] Zonnenberg I de Waal K. The definition of a haemodynamic significant duct in randomized controlled trials: a systematic literature review. Acta Paediatr. (2012) 101:247–51. 10.1111/j.1651-2227.2011.02468.x 21913976

[10] Friedman WF Hirschklau MJ Printz MP Pitlick PT Kirkpatrick SE. Pharmacologic closure of patent ductus arteriosus in the premature infant. N Engl J Med. (1976) 295:526–9. 10.1056/NEJM197609022951003 820994

[11] Mitra S Florez ID Tamayo ME Mbuagbaw L Vanniyasingam T Veroniki AA. Association of placebo, indomethacin, ibuprofen, and acetaminophen with closure of hemodynamically significant patent ductus arteriosus in preterm infants: a systematic review and meta-analysis. JAMA. (2018) 319:1221–38. 10.1001/jama.2018.1896 29584842 PMC5885871

[12] Benitz WE. Treatment of persistent patent ductus arteriosus in preterm infants: time to accept the null hypothesis? J Perinatol. (2010) 30:241–52. 10.1038/jp.2010.3 20182439

[13] Moher D Liberati A Tetzlaff J Altman DG. Preferred reporting items for systematic reviews and meta-analyses: the PRISMA statement. PLoS Med. (2009) 6:e1000097. 10.1371/journal.pmed.1000097 19621072 PMC2707599

[14] El-Mashad AE El-Mahdy H El Amrousy D Elgendy M. Comparative study of the efficacy and safety of paracetamol, ibuprofen, and indomethacin in closure of patent ductus arteriosus in preterm neonates. Eur J Pediatr. (2017) 176:233–40. 10.1007/s00431-016-2830-7 28004188

[15] Serghiou S Goodman SN. Random-effects meta-analysis: summarizing evidence with caveats. JAMA. (2019) 321:301–2. 10.1001/jama.2018.19684 30566189

[16] Higgins JP Altman DG Gotzsche PC Juni P Moher D Oxman AD. The Cochrane Collaboration's tool for assessing risk of bias in randomised trials. BMJ. (2011) 343:d5928. 10.1136/bmj.d5928 22008217 PMC3196245

[17] GRADE handbook for grading quality of evidence and strength of recommendations In: Schünemann H, Brozek J, Guyatt G, Oxman A, ed.: *The GRADE Working Group* (2013_: guidelinedevelopment.org/handbook (accessed August 7, 2019).

[18] Nestrud RM Hill DE Arrington RW Beard AG Dungan WT Lau PY. Indomethacin treatment in patent ductus arteriosus. A double-blind study utilizing indomethacin plasma levels. Dev Pharmacol Ther. (1980) 1:125–36. 10.1159/000455530 6765467

[19] Merritt TA Harris JP Roghmann K Wood B Campanella V Alexson C. Early closure of the patent ductus arteriosus in very low-birth-weight infants: a controlled trial. J Pediatr. (1981) 99:281–6. 10.1016/S0022-3476(81)80479-9 7019406

[20] Neu J Ariagno RL Johnson JD Pitlick PT Cohen RS Beets CL. A double blind study of the effects of oral indomethacin in preterm infants with patent ductus arteriosus who failed medical management. Pediatric Pharmacol. (1981) 1:245–9. 7346744

[21] Yanagi RM Wilson A Newfeld EA Aziz KU Hunt CE. Indomethacin treatment for symptomatic patent ductus arteriosus: a double-blind control study. Pediatrics. (1981) 67:647–52. 7019841

[22] Yeh TF Luken JA Thalji A Raval D Carr I Pildes RS. Intravenous indomethacin therapy in premature infants with persistent ductus arteriosus–a double-blind controlled study. J Pediatr. (1981) 98:137–45. 10.1016/S0022-3476(81)80560-4 7005415

[23] Mahony L Carnero V Brett C Heymann MA Clyman RI. Prophylactic indomethacin therapy for patent ductus arteriosus in very-low-birth-weight infants. N Engl J Med. (1982) 306:506–10. 10.1056/NEJM198203043060903 7035955

[24] Mullett MD Croghan TW Myerberg DZ Krall JM Neal WA. Indomethacin for closure of patent ductus arteriosus in prematures. Clin Pediatr. (1982) 21:217–20. 10.1177/000992288202100404 7039927

[25] Gersony WM Peckham GJ Ellison RC Miettinen OS Nadas AS. Effects of indomethacin in premature infants with patent ductus arteriosus: results of a national collaborative study. J Pediatr. (1983) 102:895–906. 10.1016/S0022-3476(83)80022-5 6343572

[26] Kaapa P Lanning P Koivisto M. Early closure of patent ductus arteriosus with indomethacin in preterm infants with idiopathic respiratory distress syndrome. Acta Paediatrica Scand. (1983) 72:179–84. 10.1111/j.1651-2227.1983.tb09693.x 6340412

[27] Rudd P Montanez P Hallidie-Smith K Silverman M. Indomethacin treatment for patent ductus arteriosus in very low birthweight infants: double blind trial. Arch Dis Child. (1983) 58:267–70. 10.1136/adc.58.4.267 6342542 PMC1627935

[28] Yeh TF Raval D Pyati S Pildes RS. Retinopathy of prematurity (ROP) and indomethacin therapy in premature infants with patent ductus arteriosus (PDA). Prostaglandins. (1983) 25:385–91. 10.1016/0090-6980(83)90041-2 6346399

[29] Mahony L Caldwell RL Girod DA Hurwitz RA Jansen RD Lemons JA. Indomethacin therapy on the first day of life in infants with very low birth weight. J Pediatr. (1985) 106:801–5. 10.1016/S0022-3476(85)80361-9 3998921

[30] Ment LR Duncan CC Ehrenkranz RA Kleinman CS Pitt BR Taylor KJ. Randomized indomethacin trial for prevention of intraventricular hemorrhage in very low birth weight infants. J Pediatr. (1985) 107:937–43. 10.1016/S0022-3476(85)80197-9 3906073

[31] Hammerman C Strates E Valaitis S. The silent ductus: its precursors and its aftermath. Pediatr Cardiol. (1986) 7:121–7. 10.1007/BF02424985 3468491

[32] Rennie JM Doyle J Cooke RW. Early administration of indomethacin to preterm infants. Arch Dis Child. (1986) 61:233–8. 10.1136/adc.61.3.233 3516077 PMC1777724

[33] Hammerman C Strates E Komar K Bui K. Failure of prophylactic indomethacin to improve the outcome of the very low birth weight infant. Dev Pharmacol Ther. (1987) 10:393–404. 10.1159/000457771 3677969

[34] Krueger E Mellander M Bratton D Cotton R. Prevention of symptomatic patent ductus arteriosus with a single dose of indomethacin. J Pediatr. (1987) 111:749–54. 10.1016/S0022-3476(87)80262-7 3312552

[35] Vincer M Allen A Evans J Nwaesei C Stinson D Rees E. Early intravenous indomethacin prolongs respiratory support in very low birth weight infants. Acta Paediatrica Scand. (1987) 76:894–7. 10.1111/j.1651-2227.1987.tb17260.x 3321891

[36] Weesner KM Dillard RG Boyle RJ Block SM. Prophylactic treatment of asymptomatic patent ductus arteriosus in premature infants with respiratory distress syndrome. Southern Med J. (1987) 80:706–8. 10.1097/00007611-198706000-00010 3296225

[37] Bandstra ES Montalvo BM Goldberg RN Pacheco I Ferrer PL Flynn J. Prophylactic indomethacin for prevention of intraventricular hemorrhage in premature infants. Pediatrics. (1988) 82:533–42. 3174314

[38] Hanigan WC Kennedy G Roemisch F Anderson R Cusack T Powers W. Administration of indomethacin for the prevention of periventricular-intraventricular hemorrhage in high-risk neonates. J Pediatr. (1988) 112:941–7. 10.1016/S0022-3476(88)80224-5 3373404

[39] Ment LR Duncan CC Ehrenkranz RA Kleinman CS Taylor KJ Scott DT. Randomized low-dose indomethacin trial for prevention of intraventricular hemorrhage in very low birth weight neonates. J Pediatr. (1988) 112:948–55. 10.1016/S0022-3476(88)80225-7 3373405

[40] Lai TH Soong WJ Hwang B. Indomethacin for the prevention of symptomatic patent ductus arteriosus in very low birth weight infants. Zhonghua Min Guo Xiao Er Ke Yi Xue Hui Za Zhi. (1990) 31:17–23. 2278224

[41] Ment LR Oh W Ehrenkranz RA Philip AG Vohr B Allan W. Low-dose indomethacin and prevention of intraventricular hemorrhage: a multicenter randomized trial. Pediatrics. (1994) 93:543–50. 8134206

[42] Couser RJ Ferrara TB Wright GB Cabalka AK Schilling CG Hoekstra RE. Prophylactic indomethacin therapy in the first twenty-four hours of life for the prevention of patent ductus arteriosus in preterm infants treated prophylactically with surfactant in the delivery room. J Pediatr. (1996) 128(5 Pt 1):631–7. 10.1016/S0022-3476(96)80127-2 8627434

[43] Supapannachart S Khowsathit P Patchakapati B. Indomethacin prophylaxis for patent ductus arteriosus (PDA) in infants with a birth weight of less than 1250 grams. J Med Assoc Thai. (1999) 82(Suppl. 1):S87–92. 10730525

[44] Couser RJ Hoekstra RE Ferrara TB Wright GB Cabalka AK Connett JE. Neurodevelopmental follow-up at 36 months' corrected age of preterm infants treated with prophylactic indomethacin. Arch Pediatr Adolesc Med. (2000) 154:598–602. 10.1001/archpedi.154.6.598 10850507

[45] De Carolis MP Romagnoli C Polimeni V Piersigilli F Zecca E Papacci P. Prophylactic ibuprofen therapy of patent ductus arteriosus in preterm infants. Eur J Pediatr. (2000) 159:364–8. 10.1007/s004310051288 10834523

[46] Schmidt B Davis P Moddemann D Ohlsson A Roberts RS Saigal S. Long-term effects of indomethacin prophylaxis in extremely-low-birth-weight infants. N Engl J Med. (2001) 344:1966–72. 10.1056/NEJM200106283442602 11430325

[47] Osborn DA Evans N Kluckow M. Effect of early targeted indomethacin on the ductus arteriosus and blood flow to the upper body and brain in the preterm infant. Arch Dis Child Fetal Neonatal Ed. (2003) 88:F477–82. 10.1136/fn.88.6.F477 14602694 PMC1763239

[48] Gournay V Roze JC Kuster A Daoud P Cambonie G Hascoet JM. Prophylactic ibuprofen versus placebo in very premature infants: a randomised, double-blind, placebo-controlled trial. Lancet. (2004) 364:1939–44. 10.1016/S0140-6736(04)17476-X 15567009

[49] Van Overmeire B Allegaert K Casaer A Debauche C Decaluwe W Jespers A. Prophylactic ibuprofen in premature infants: a multicentre, randomised, double-blind, placebo-controlled trial. Lancet. (2004) 364:1945–9. 10.1016/S0140-6736(04)17477-1 15567010

[50] Dani C Bertini G Pezzati M Poggi C Guerrini P Martano C. Prophylactic ibuprofen for the prevention of intraventricular hemorrhage among preterm infants: a multicenter, randomized study. Pediatrics. (2005) 115:1529–35. 10.1542/peds.2004-1178 15930213

[51] Sangtawesin V Sangtawesin C Raksasinborisut C Sathirakul K Kanjanapattanakul W Khorana M. Oral ibuprofen prophylaxis for symptomatic patent ductus arteriosus of prematurity. J Med Assoc Thai. (2006) 89:314–21. 16696414

[52] Sangtawesin C Sangtawesin V Lertsutthiwong W Kanjanapattanakul W Khorana M Ayudhaya JK. Prophylaxis of symptomatic patent ductus arteriosus with oral ibuprofen in very low birth weight infants. J Med Assoc Thai. (2008) 91(Suppl. 3):S28–34. 19255990

[53] Aranda JV Clyman R Cox B Van Overmeire B Wozniak P Sosenko I. A randomized, double-blind, placebo-controlled trial on intravenous ibuprofen L-lysine for the early closure of nonsymptomatic patent ductus arteriosus within 72 hours of birth in extremely low-birth-weight infants. Am J Perinatol. (2009) 26:235–45. 10.1055/s-0028-1103515 19067286

[54] Amoozgar H Ghodstehrani M Pishva N. Oral ibuprofen and ductus arteriosus closure in full-term neonates: a prospective case-control study. Pediatr Cardiol. (2010) 31:40–3. 10.1007/s00246-009-9542-y 19841966

[55] Maruyama K Fujiu T. Effects of prophylactic indomethacin on renal and intestinal blood flows in premature infants. Pediatr Int. (2012) 54:480–5. 10.1111/j.1442-200X.2012.03583.x 22348233

[56] Sosenko IR Fajardo MF Claure N Bancalari E. Timing of patent ductus arteriosus treatment and respiratory outcome in premature infants: a double-blind randomized controlled trial. J Pediatr. (2012) 160:929–35.e921. 10.1016/j.jpeds.2011.12.031 22284563

[57] Bagnoli F Rossetti A Messina G Mori A Casucci M Tomasini B. Treatment of patent ductus arteriosus (PDA) using ibuprofen: renal side-effects in VLBW and ELBW newborns. J Matern Fetal Neonatal Med. (2013) 26:423–9. 10.3109/14767058.2012.733775 23057804

[58] Kanmaz G Erdeve O Canpolat FE Oguz SS Uras N Altug N. Serum ibuprofen levels of extremely preterm infants treated prophylactically with oral ibuprofen to prevent patent ductus arteriosus. Eur J Clin Pharmacol. (2013) 69:1075–81. 10.1007/s00228-012-1438-8 23128963

[59] Ding YJ Han B Yang B Zhu M. NT-proBNP plays an important role in the effect of ibuprofen on preterm infants with patent ductus arteriosus. Eur Rev Med Pharmacol Sci. (2014) 18:2596–8. 25317790

[60] Jannatdoust A Samadi M Yeganehdoust S Heydarzadeh M Alikhah H Piri R. Effects of intravenous indomethacin on reduction of symptomatic patent ductus arteriosus cases and decreasing the need for prolonged mechanical ventilation. J Cardiovasc Thorac Res. (2014) 6:257–9. 10.15171/jcvtr.2014.022 25610559 PMC4291606

[61] Kluckow M Jeffery M Gill A Evans N. A randomised placebo-controlled trial of early treatment of the patent ductus arteriosus. Arch Dis Child Fetal Neonatal Ed. (2014) 99:F99–104. 10.1136/archdischild-2013-304695 24317704

[62] Harkin P Harma A Aikio O Valkama M Leskinen M Saarela T. Paracetamol accelerates closure of the ductus arteriosus after premature birth: a randomized trial. J Pediatr. (2016) 177:72–7 e72. 10.1016/j.jpeds.2016.04.066 27215779

[63] Clyman RI Liebowitz M Kaempf J Erdeve O Bulbul A Hakansson S. PDA-TOLERATE trial: an exploratory randomized controlled trial of treatment of moderate-to-large patent ductus arteriosus at 1 week of age. J Pediatr. (2019) 205:41–8 e46. 10.1016/j.jpeds.2018.09.012 30340932 PMC6502709

[64] Juujarvi S Kallankari H Patsi P Leskinen M Saarela T Hallman M. Follow-up study of the early, randomised paracetamol trial to preterm infants, found no adverse reactions at the two-years corrected age. Acta Paediatr. (2019) 108:452–8. 10.1111/apa.14614 30325529

[65] Fowlie PW Davis PG McGuire W. Prophylactic intravenous indomethacin for preventing mortality and morbidity in preterm infants. Cochrane Database Syst Rev. (2010) 2010:CD000174. 10.1002/14651858.CD000174.pub2 20614421 PMC7045285

[66] Keating P Verhagen E van Hoften J ter Horst H Bos AF. Effect of indomethacin infused over 30 minutes on cerebral fractional tissue oxygen extraction in preterm newborns with a patent ductus arteriosus. Neonatology. (2010) 98:232–7. 10.1159/000283946 20389128

[67] Patel J Roberts I Azzopardi D Hamilton P Edwards AD. Randomized double-blind controlled trial comparing the effects of ibuprofen with indomethacin on cerebral hemodynamics in preterm infants with patent ductus arteriosus. Pediatr Res. (2000) 47:36–42. 10.1203/00006450-200001000-00009 10625080

[68] Khanafer-Larocque I Soraisham A Stritzke A Al Awad E Thomas S Murthy P. Intraventricular hemorrhage: risk factors and association with patent ductus arteriosus treatment in extremely preterm neonates. Front Pediatr. (2019) 7:408. 10.3389/fped.2019.00408 31696098 PMC6817605

[69] Nelin TD Pena E Giacomazzi T Lee S Logan JW Moallem M. Outcomes following indomethacin prophylaxis in extremely preterm infants in an all-referral NICU. J Perinatol. (2017) 37:932–7. 10.1038/jp.2017.71 28617424

[70] Roberts D Brown J Medley N Dalziel SR. Antenatal corticosteroids for accelerating fetal lung maturation for women at risk of preterm birth. Cochrane Database Syst Rev. (2017) 3:Cd004454. 10.1002/14651858.CD004454.pub3 28321847 PMC6464568

[71] Mukerji A Shah V Shah PS. Periventricular/intraventricular hemorrhage and neurodevelopmental outcomes: a meta-analysis. Pediatrics. (2015) 136:1132–43. 10.1542/peds.2015-0944 26598455

[72] Harkin P Marttila R Pokka T Saarela T Hallman M. Morbidities associated with patent ductus arteriosus in preterm infants. Nationwide cohort study. J Maternal-Fetal Neonatal Med. (2018) 31:2576–83. 10.1080/14767058.2017.1347921 28651469

[73] Mohamed MA El-Dib M Alqahtani S Alyami K Ibrahim AN Aly H. Patent ductus arteriosus in premature infants: to treat or not to treat? J Perinatol. (2017) 37:652–7. 10.1038/jp.2017.4 28206995

[74] Ohlsson A Walia R Shah SS Ibuprofen for the treatment of patent ductus arteriosus in preterm or low birth weight (or both) infants. Cochrane Database Syst Rev. (2018) 9:Cd003481 10.1002/14651858.CD003481.pub7 30264852 PMC6513618

[75] Jansen EJS Dijkman KP van Lingen RA de Vries WB Vijlbrief DC de Boode WP. Using benchmarking to identify inter-centre differences in persistent ductus arteriosus treatment: can we improve outcome? Cardiol Young. (2017) 27:1488–96. 10.1017/S104795111700052X 28399954

