# Supplementary material for: Factors Associated With Benefit of Treatment of Patent Ductus Arteriosus in Preterm Infants: A Systematic Review and Meta-Analysis
Source: Front Pediatr. 2021 Feb 9;9:626262. doi: 10.3389/fped.2021.626262 (PMC7899974; doi:10.3389/fped.2021.626262)
Supplement: Supplementary file 1 [file Data_Sheet_1.docx]

**List of supplements**

1. Search Strategy page 2
2. GRADE evidence profile page 3-20
3. PRISMA checklist page 21-22

**Supplement 1**

**Search Strategy**

(infant*[tiab] OR newborn*[tiab] OR neonate*[tiab]) AND (preterm[tiab] OR premature[tiab] OR immature[tiab]) OR "Infant, Premature"[Mesh]) AND (((ductus[tiab] OR arteriosus[tiab] OR Botalli[tiab])) OR "Ductus Arteriosus, Patent"[Mesh]) OR "Ductus Arteriosus"[Mesh]

**Supplement 2**

**GRADE evidence profile**

Failure to close a patent ductus arteriosus (PDA)

**Author(s)**: EJS Jansen, T Hundscheid, W Onland, EMW Kooi, P Andriessen, WP de Boode

**Date**: 2019-09-10

**Question**: Should pharmacological treatment versus placebo/no treatment be used in preterm infants with a PDA to prevent failure to close this PDA?

**Setting**: Preterm infants with a PDA

| **Certainty assessment** | | | | | | | **№ of patients** | | **Effect** | | **Certainty** | **Importance** |
| --- | --- | --- | --- | --- | --- | --- | --- | --- | --- | --- | --- | --- |
| **№ of studies** | **Study design** | **Risk of bias** | **Inconsistency** | **Indirectness** | **Imprecision** | **Other considerations** | **pharmacological treatment** | **placebo/no treatment** | **Relative (95% CI)** | **Absolute (95% CI)** |  |  |
| **Failure to close a patent ductus arteriosus** | | | | | | | | | | | | |
| 40 | randomised trials | serious ^a^ | serious ^b^ | serious ^c^ | not serious | publication bias strongly suspected ^d^ | 417/2065 (20.2%) | 1113/2226 (50.0%) | **RR 0.40** (0.33 to 0.48) | **300 fewer per 1.000** (from 335 fewer to 260 fewer) | ⨁◯◯◯ VERY LOW | CRITICAL |
| **Failure close PDA - birthweight - BW <1000 gram** | | | | | | | | | | | | |
| 13 | randomised trials | serious ^e^ | serious ^b^ | not serious | not serious | publication bias strongly suspected ^d^ | 221/1020 (21.7%) | 476/1025 (46.4%) | **RR 0.48** (0.37 to 0.63) | **241 fewer per 1.000** (from 293 fewer to 172 fewer) | ⨁◯◯◯ VERY LOW | CRITICAL |
| **Failure close PDA - birthweight - BW 1000-1250 gram** | | | | | | | | | | | | |
| 18 | randomised trials | serious ^a^ | serious ^b^ | not serious | not serious | publication bias strongly suspected ^d^ | 151/751 (20.1%) | 369/765 (48.2%) | **RR 0.37** (0.27 to 0.51) | **304 fewer per 1.000** (from 352 fewer to 236 fewer) | ⨁◯◯◯ VERY LOW | CRITICAL |
| **Failure close PDA - birthweight - BW 1251-1500 gram** | | | | | | | | | | | | |
| 5 | randomised trials | not serious | not serious | not serious | serious ^f^ | none | 10/101 (9.9%) | 37/103 (35.9%) | **RR 0.32** (0.16 to 0.65) | **244 fewer per 1.000** (from 302 fewer to 126 fewer) | ⨁⨁⨁◯ MODERATE | CRITICAL |
| **Failure close PDA - birthweight - BW >1500** | | | | | | | | | | | | |
| 1 | randomised trials | serious ^g^ | not serious | not serious | serious ^f^ | none | 1/13 (7.7%) | 7/14 (50.0%) | **RR 0.15** (0.02 to 1.09) | **425 fewer per 1.000** (from 490 fewer to 45 more) | ⨁⨁◯◯ LOW | IMPORTANT |
| **Failure close PDA- gestational age - GA < 28 weeks** | | | | | | | | | | | | |
| 9 | randomised trials | serious ^h^ | serious ^b^ | not serious | serious ^i^ | publication bias strongly suspected ^d^ | 258/1021 (25.3%) | 480/1018 (47.2%) | **RR 0.63** (0.47 to 0.84) | **174 fewer per 1.000** (from 250 fewer to 75 fewer) | ⨁◯◯◯ VERY LOW | CRITICAL |
| **Failure close PDA- gestational age - GA 28-32 weeks** | | | | | | | | | | | | |
| 29 | randomised trials | serious ^a^ | not serious | not serious | not serious | publication bias strongly suspected ^d^ | 127/899 (14.1%) | 428/924 (46.3%) | **RR 0.34** (0.28 to 0.41) | **306 fewer per 1.000** (from 334 fewer to 273 fewer) | ⨁⨁◯◯ LOW | CRITICAL |
| **Failure close PDA - PNA - PNA <24** | | | | | | | | | | | | |
| 24 | randomised trials | serious ^e^ | serious ^b^ | not serious | not serious | publication bias strongly suspected ^d^ | 302/1526 (19.8%) | 718/1549 (46.4%) | **RR 0.40** (0.31 to 0.51) | **278 fewer per 1.000** (from 320 fewer to 227 fewer) | ⨁◯◯◯ VERY LOW | CRITICAL |
| **Failure close PDA - PNA - PNA 24-72h** | | | | | | | | | | | | |
| 2 | randomised trials | not serious | not serious | not serious | serious ^f^ | none | 1/35 (2.9%) | 11/33 (33.3%) | **RR 0.12** (0.02 to 0.63) | **293 fewer per 1.000** (from 327 fewer to 123 fewer) | ⨁⨁⨁◯ MODERATE | CRITICAL |
| **Failure close PDA - PNA - PNA >72h** | | | | | | | | | | | | |
| 13 | randomised trials | serious ^j^ | serious ^b^ | not serious | not serious | publication bias strongly suspected ^d^ | 113/469 (24.1%) | 376/607 (61.9%) | **RR 0.41** (0.30 to 0.57) | **365 fewer per 1.000** (from 434 fewer to 266 fewer) | ⨁◯◯◯ VERY LOW | CRITICAL |
| **Failure to close PDA - open label treatment - open label treatment <25%** | | | | | | | | | | | | |
| 6 | randomised trials | serious ^k^ | not serious | serious ^c^ | serious ^l^ | publication bias strongly suspected ^d^ | 47/374 (12.6%) | 138/388 (35.6%) | **RR 0.37** (0.27 to 0.50) | **224 fewer per 1.000** (from 260 fewer to 178 fewer) | ⨁◯◯◯ VERY LOW | CRITICAL |
| **Failure to close PDA - open label treatment - open label treatment 25-50%** | | | | | | | | | | | | |
| 14 | randomised trials | serious ^h^ | not serious | serious ^c^ | not serious | publication bias strongly suspected ^d^ | 225/1160 (19.4%) | 529/1163 (45.5%) | **RR 0.40** (0.31 to 0.52) | **273 fewer per 1.000** (from 314 fewer to 218 fewer) | ⨁◯◯◯ VERY LOW | IMPORTANT |
| **Failure to close PDA - open label treatment - open label treatment >50%** | | | | | | | | | | | | |
| 13 | randomised trials | serious ^m^ | serious ^b^ | serious ^c^ | serious ^i^ | publication bias strongly suspected ^d^ | 101/350 (28.9%) | 350/493 (71.0%) | **RR 0.40** (0.25 to 0.62) | **426 fewer per 1.000** (from 532 fewer to 270 fewer) | ⨁◯◯◯ VERY LOW | IMPORTANT |
| **Failure to close PDA - risk of bias - Low risk** | | | | | | | | | | | | |
| 18 | randomised trials | not serious | serious ^b^ | serious ^c^ | not serious | publication bias strongly suspected ^d^ | 295/1444 (20.4%) | 825/1605 (51.4%) | **RR 0.39** (0.30 to 0.50) | **314 fewer per 1.000** (from 360 fewer to 257 fewer) | ⨁◯◯◯ VERY LOW | CRITICAL |
| **Failure to close PDA - risk of bias - Intermediate risk** | | | | | | | | | | | | |
| 17 | randomised trials | serious ^a^ | serious ^b^ | serious ^c^ | not serious | publication bias strongly suspected ^d^ | 99/462 (21.4%) | 244/465 (52.5%) | **RR 0.37** (0.26 to 0.53) | **331 fewer per 1.000** (from 388 fewer to 247 fewer) | ⨁◯◯◯ VERY LOW | CRITICAL |
| **Failure to close PDA - risk of bias - High risk** | | | | | | | | | | | | |
| 5 | randomised trials | serious ^e^ | serious ^b^ | serious ^c^ | serious ^f^ | publication bias strongly suspected ^d^ | 23/159 (14.5%) | 44/156 (28.2%) | **RR 0.42** (0.19 to 0.97) | **164 fewer per 1.000** (from 228 fewer to 8 fewer) | ⨁◯◯◯ VERY LOW | IMPORTANT |

**CI:** Confidence interval; **RR:** Risk ratio

#### Explanations

a. Selection bias, no blinding of personnel, incomplete outcome data, or reporting bias

b. Heterogeneity > 50

c. Preterm infants with differences in birthweight, gestational age, and start of treatment

d. Asymmetrical funnel plot

e. Selection bias, no blinding of personnel, or incomplete outcome data

f. Number of events <300 and wide CI

g. No blinding of personnel

h. No blinding of personnel or incomplete outcome data

i. Wide CI

j. No blinding of personnel, incomplete outcome data, or reporting bias

k. Selection bias or incomplete outcome data

l. Number of events < 300

m. Selection bias, no blinding of personnel, or reporting bias

Need for surgical ligation in case of a patent ductus arteriosus (PDA)

**Author(s)**: EJS Jansen, T Hundscheid, W Onland, EMW Kooi, P Andriessen, WP de Boode

**Date**: 2019-09-10

**Question**: Should pharmacological treatment versus placebo/no treatment be used in preterm infants with a PDA to prevent need for surgical ligation?

**Setting**: Preterm infants with a PDA

| **Certainty assessment** | | | | | | | **№ of patients** | | **Effect** | | **Certainty** | **Importance** |
| --- | --- | --- | --- | --- | --- | --- | --- | --- | --- | --- | --- | --- |
| **№ of studies** | **Study design** | **Risk of bias** | **Inconsistency** | **Indirectness** | **Imprecision** | **Other considerations** | **pharmacological treatment** | **placebo/no treatment** | **Relative (95% CI)** | **Absolute (95% CI)** |  |  |
| **Need for surgical ligation** | | | | | | | | | | | | |
| 24 | randomised trials | serious ^a^ | not serious | serious ^b^ | serious ^c^ | none | 108/1634 (6.6%) | 182/1655 (11.0%) | **RR 0.61** (0.49 to 0.76) | **43 fewer per 1.000** (from 56 fewer to 26 fewer) | ⨁◯◯◯ VERY LOW | CRITICAL |
| **Need ligation - birthweight - BW <1000 gram** | | | | | | | | | | | | |
| 11 | randomised trials | serious ^d^ | not serious | not serious | serious ^e^ | none | 71/1051 (6.8%) | 117/1051 (11.1%) | **RR 0.61** (0.46 to 0.80) | **43 fewer per 1.000** (from 60 fewer to 22 fewer) | ⨁⨁◯◯ LOW | CRITICAL |
| **Need ligation - birthweight - BW 1000-1250 gram** | | | | | | | | | | | | |
| 10 | randomised trials | serious ^f^ | serious ^g^ | not serious | serious ^e^ | publication bias strongly suspected ^h^ | 34/537 (6.3%) | 59/551 (10.7%) | **RR 0.60** (0.41 to 0.89) | **43 fewer per 1.000** (from 63 fewer to 12 fewer) | ⨁◯◯◯ VERY LOW | CRITICAL |
| **Need ligation - birthweight - BW 1251-1500 gram** | | | | | | | | | | | | |
| 2 | randomised trials | serious ^i^ | not serious | not serious | serious ^e^ | none | 1/36 (2.8%) | 1/39 (2.6%) | **RR 1.08** (0.08 to 15.46) | **2 more per 1.000** (from 24 fewer to 371 more) | ⨁⨁◯◯ LOW | CRITICAL |
| **Need ligation - gestational age - GA < 28 weeks** | | | | | | | | | | | | |
| 9 | randomised trials | serious ^j^ | not serious | not serious | serious ^e^ | publication bias strongly suspected ^h^ | 82/1065 (7.7%) | 122/1065 (11.5%) | **RR 0.67** (0.52 to 0.87) | **38 fewer per 1.000** (from 55 fewer to 15 fewer) | ⨁◯◯◯ VERY LOW | CRITICAL |
| **Need ligation - gestational age - GA 28-32 weeks** | | | | | | | | | | | | |
| 13 | randomised trials | serious ^k^ | not serious | not serious | serious ^e^ | publication bias strongly suspected ^h^ | 23/547 (4.2%) | 54/563 (9.6%) | **RR 0.45** (0.29 to 0.71) | **53 fewer per 1.000** (from 68 fewer to 28 fewer) | ⨁◯◯◯ VERY LOW | CRITICAL |
| **Need ligation - PNA - PNA <24** | | | | | | | | | | | | |
| 14 | randomised trials | serious ^d^ | not serious | not serious | serious ^e^ | publication bias strongly suspected ^h^ | 57/1286 (4.4%) | 113/1308 (8.6%) | **RR 0.52** (0.39 to 0.71) | **41 fewer per 1.000** (from 53 fewer to 25 fewer) | ⨁◯◯◯ VERY LOW | CRITICAL |
| **Need ligation - PNA - PNA 24-72h** | | | | | | | | | | | | |
| 2 | randomised trials | serious ^d^ | not serious | not serious | serious ^e^ | none | 9/66 (13.6%) | 11/64 (17.2%) | **RR 0.78** (0.35 to 1.76) | **38 fewer per 1.000** (from 112 fewer to 131 more) | ⨁⨁◯◯ LOW | CRITICAL |
| **Need ligation - PNA - PNA >72h** | | | | | | | | | | | | |
| 8 | randomised trials | serious ^f^ | serious ^g^ | not serious | serious ^e^ | none | 42/282 (14.9%) | 58/283 (20.5%) | **RR 0.75** (0.52 to 1.07) | **51 fewer per 1.000** (from 98 fewer to 14 more) | ⨁◯◯◯ VERY LOW | CRITICAL |
| **Need ligation - open label treatment - open label treatment <25%** | | | | | | | | | | | | |
| 3 | randomised trials | serious ^l^ | not serious | not serious | serious ^e^ | publication bias strongly suspected ^h^ | 6/271 (2.2%) | 16/278 (5.8%) | **RR 0.39** (0.15 to 0.97) | **35 fewer per 1.000** (from 49 fewer to 2 fewer) | ⨁◯◯◯ VERY LOW | CRITICAL |
| **Need ligation - open label treatment - open label treatment 25-50%** | | | | | | | | | | | | |
| 11 | randomised trials | serious ^j^ | not serious | serious ^b^ | serious ^e^ | publication bias strongly suspected ^h^ | 59/1108 (5.3%) | 116/1118 (10.4%) | **RR 0.52** (0.39 to 0.70) | **50 fewer per 1.000** (from 63 fewer to 31 fewer) | ⨁◯◯◯ VERY LOW | IMPORTANT |
| **Need ligation - open label treatment - open label treatment >50%** | | | | | | | | | | | | |
| 8 | randomised trials | serious ^a^ | not serious | serious ^b^ | serious ^e^ | publication bias strongly suspected ^h^ | 25/178 (14.0%) | 41/183 (22.4%) | **RR 0.63** (0.40 to 0.99) | **83 fewer per 1.000** (from 134 fewer to 2 fewer) | ⨁◯◯◯ VERY LOW | IMPORTANT |
| **Need ligation- risk of bias - Low risk** | | | | | | | | | | | | |
| 13 | randomised trials | not serious | not serious | serious ^b^ | serious ^e^ | publication bias strongly suspected ^h^ | 72/1263 (5.7%) | 137/1285 (10.7%) | **RR 0.55** (0.42 to 0.71) | **48 fewer per 1.000** (from 62 fewer to 31 fewer) | ⨁◯◯◯ VERY LOW | CRITICAL |
| **Need ligation- risk of bias - Intermediate risk** | | | | | | | | | | | | |
| 8 | randomised trials | serious ^a^ | serious ^g^ | serious ^b^ | serious ^e^ | none | 23/241 (9.5%) | 31/242 (12.8%) | **RR 0.74** (0.46 to 1.20) | **33 fewer per 1.000** (from 69 fewer to 26 more) | ⨁◯◯◯ VERY LOW | CRITICAL |
| **Need ligation- risk of bias - High risk** | | | | | | | | | | | | |
| 3 | randomised trials | serious ^d^ | not serious | serious ^b^ | serious ^e^ | none | 13/130 (10.0%) | 14/128 (10.9%) | **RR 0.90** (0.45 to 1.82) | **11 fewer per 1.000** (from 60 fewer to 90 more) | ⨁◯◯◯ VERY LOW | IMPORTANT |

**CI:** Confidence interval; **RR:** Risk ratio

#### Explanations

a. Selection bias, no blinding of personnel, incomplete outcome data, or reporting bias

b. Preterm infants with differences in birthweight, gestational age, and start of treatment

c. Number of events <300

d. Selection bias, no blinding of personnel, or incomplete outcome data

e. Number of events <300 and wide CI

f. No blinding of personnel or reporting bias

g. Heterogeneity > 50

h. Asymmetrical funnel plot

i. Selection bias, no blinding of personnel, or incomplete outcome data

j. No blinding of personnel or incomplete outcome data

k. Selection bias, no blinding of personnel, or reporting bias

l. Selection bias

Necrotizing enterocolitis (NEC)

**Author(s)**: EJS Jansen, T Hundscheid, W Onland, EMW Kooi, P Andriessen, WP de Boode

**Date**: 2019-09-10

**Question**: Should pharmacological treatment versus placebo/no treatment be used in preterm infants with a PDA to prevent necrotizing enterocolitis?

**Setting**: Preterm infants with a PDA

| **Certainty assessment** | | | | | | | **№ of patients** | | **Effect** | | **Certainty** | **Importance** |
| --- | --- | --- | --- | --- | --- | --- | --- | --- | --- | --- | --- | --- |
| **№ of studies** | **Study design** | **Risk of bias** | **Inconsistency** | **Indirectness** | **Imprecision** | **Other considerations** | **pharmacological treatment** | **placebo/no treatment** | **Relative (95% CI)** | **Absolute (95% CI)** |  |  |
| **Necrotizing enterocolitis (any definition)** | | | | | | | | | | | | |
| 29 | randomised trials | serious ^a^ | not serious | serious ^b^ | serious ^c^ | none | 155/1831 (8.5%) | 140/1855 (7.5%) | **RR 1.10** (0.89 to 1.37) | **8 more per 1.000** (from 8 fewer to 28 more) | ⨁◯◯◯ VERY LOW | CRITICAL |
| **NEC- birthweight - BW <1000 gram** | | | | | | | | | | | | |
| 14 | randomised trials | serious ^a^ | not serious | not serious | serious ^c^ | none | 115/1163 (9.9%) | 106/1167 (9.1%) | **RR 1.07** (0.84 to 1.37) | **6 more per 1.000** (from 15 fewer to 34 more) | ⨁⨁◯◯ LOW | CRITICAL |
| **NEC- birthweight - BW 1000-1250 gram** | | | | | | | | | | | | |
| 13 | randomised trials | serious ^d^ | not serious | not serious | serious ^c^ | none | 31/637 (4.9%) | 26/654 (4.0%) | **RR 1.21** (0.74 to 1.97) | **8 more per 1.000** (from 10 fewer to 39 more) | ⨁⨁◯◯ LOW | CRITICAL |
| **NEC- birthweight - BW 1251-1500 gram** | | | | | | | | | | | | |
| 1 | randomised trials | not serious | not serious | not serious | serious ^c^ | none | 8/21 (38.1%) | 6/20 (30.0%) | **RR 1.27** (0.54 to 3.01) | **81 more per 1.000** (from 138 fewer to 603 more) | ⨁⨁⨁◯ MODERATE | CRITICAL |
| **NEC- gestational age - GA < 28 weeks** | | | | | | | | | | | | |
| 12 | randomised trials | serious ^e^ | not serious | not serious | serious ^c^ | publication bias strongly suspected ^f^ | 124/1185 (10.5%) | 102/1182 (8.6%) | **RR 1.20** (0.94 to 1.54) | **17 more per 1.000** (from 5 fewer to 47 more) | ⨁◯◯◯ VERY LOW | CRITICAL |
| **NEC- gestational age - GA 28-32 weeks** | | | | | | | | | | | | |
| 16 | randomised trials | serious ^a^ | not serious | not serious | serious ^c^ | none | 30/636 (4.7%) | 36/659 (5.5%) | **RR 0.86** (0.56 to 1.34) | **8 fewer per 1.000** (from 24 fewer to 19 more) | ⨁⨁◯◯ LOW | CRITICAL |
| **NEC- PNA - PNA <24** | | | | | | | | | | | | |
| 21 | randomised trials | serious ^a^ | not serious | not serious | serious ^c^ | none | 111/1502 (7.4%) | 106/1529 (6.9%) | **RR 1.05** (0.82 to 1.35) | **3 more per 1.000** (from 12 fewer to 24 more) | ⨁⨁◯◯ LOW | CRITICAL |
| **NEC- PNA - PNA 24-72h** | | | | | | | | | | | | |
| 2 | randomised trials | not serious | not serious | not serious | serious ^c^ | none | 13/75 (17.3%) | 8/71 (11.3%) | **RR 1.54** (0.72 to 3.32) | **61 more per 1.000** (from 32 fewer to 261 more) | ⨁⨁⨁◯ MODERATE | CRITICAL |
| **NEC- PNA - PNA >72h** | | | | | | | | | | | | |
| 7 | randomised trials | not serious | not serious | not serious | serious ^c^ | none | 43/452 (9.5%) | 32/455 (7.0%) | **RR 1.33** (0.87 to 2.04) | **23 more per 1.000** (from 9 fewer to 73 more) | ⨁⨁⨁◯ MODERATE | CRITICAL |
| **NEC- crossover - crossover <25%** | | | | | | | | | | | | |
| 5 | randomised trials | serious ^g^ | not serious | serious ^h^ | serious ^c^ | publication bias strongly suspected ^f^ | 10/321 (3.1%) | 17/337 (5.0%) | **RR 0.65** (0.31 to 1.33) | **18 fewer per 1.000** (from 35 fewer to 17 more) | ⨁◯◯◯ VERY LOW | CRITICAL |
| **NEC- crossover - crossover 25-50%** | | | | | | | | | | | | |
| 12 | randomised trials | serious ^a^ | not serious | serious ^b^ | serious ^c^ | publication bias strongly suspected ^f^ | 118/1187 (9.9%) | 108/1192 (9.1%) | **RR 1.09** (0.85 to 1.39) | **8 more per 1.000** (from 14 fewer to 35 more) | ⨁◯◯◯ VERY LOW | IMPORTANT |
| **NEC- crossover - crossover >50%** | | | | | | | | | | | | |
| 10 | randomised trials | serious ^d^ | not serious | serious ^b^ | serious ^c^ | none | 18/232 (7.8%) | 14/235 (6.0%) | **RR 1.24** (0.68 to 2.28) | **14 more per 1.000** (from 19 fewer to 76 more) | ⨁◯◯◯ VERY LOW | IMPORTANT |
| **NEC- risk of bias - Low risk** | | | | | | | | | | | | |
| 15 | randomised trials | not serious | not serious | serious ^b^ | serious ^c^ | none | 105/1372 (7.7%) | 102/1395 (7.3%) | **RR 1.04** (0.80 to 1.35) | **3 more per 1.000** (from 15 fewer to 26 more) | ⨁⨁◯◯ LOW | CRITICAL |
| **NEC- risk of bias - Intermediate risk** | | | | | | | | | | | | |
| 11 | randomised trials | serious ^a^ | not serious | serious ^b^ | serious ^c^ | none | 33/326 (10.1%) | 19/329 (5.8%) | **RR 1.61** (0.98 to 2.64) | **35 more per 1.000** (from 1 fewer to 95 more) | ⨁◯◯◯ VERY LOW | CRITICAL |
| **NEC- risk of bias - High risk** | | | | | | | | | | | | |
| 3 | randomised trials | serious ^d^ | not serious | serious ^b^ | serious ^c^ | none | 17/133 (12.8%) | 19/131 (14.5%) | **RR 0.84** (0.47 to 1.53) | **23 fewer per 1.000** (from 77 fewer to 77 more) | ⨁◯◯◯ VERY LOW | IMPORTANT |

**CI:** Confidence interval; **RR:** Risk ratio

#### Explanations

a. Selection bias, no blinding of personnel or incomplete outcome data

b. Preterm infants with differences in birthweight, gestational age and start of treatment

c. Number of events <300 and wide CI

d. Selection bias or no blinding of personnel

e. No blinding of personnel or incomplete outcome data

f. Asymmetrical funnelplot

g. Selection bias or incomplete outcome date

h. Preterm infants with differences in birthweight

Bronchopulmonary dysplasia (BPD)

**Author(s)**: EJS Jansen, T Hundscheid, W Onland, EMW Kooi, P Andriessen, WP de Boode

**Date**: 2019-09-10

**Question**: Should pharmacological treatment versus placebo/no treatment be used in preterm infants with a PDA to prevent bronchopulmonary bronchopulmonary dysplasia?

**Setting**: Preterm infants with a PDA

| **Certainty assessment** | | | | | | | **№ of patients** | | **Effect** | | **Certainty** | **Importance** |
| --- | --- | --- | --- | --- | --- | --- | --- | --- | --- | --- | --- | --- |
| **№ of studies** | **Study design** | **Risk of bias** | **Inconsistency** | **Indirectness** | **Imprecision** | **Other considerations** | **pharmacological treatment** | **placebo/no treatment** | **Relative (95% CI)** | **Absolute (95% CI)** |  |  |
| **Bronchopulmonary disease (any definition)** | | | | | | | | | | | | |
| 26 | randomised trials | serious ^a^ | serious ^b^ | serious ^c^ | not serious | publication bias strongly suspected ^d^ | 624/1511 (41.3%) | 623/1494 (41.7%) | **RR 0.97** (0.85 to 1.10) | **13 fewer per 1.000** (from 63 fewer to 42 more) | ⨁◯◯◯ VERY LOW | CRITICAL |
| **BPD - birthweight - BW <1000 gram** | | | | | | | | | | | | |
| 13 | randomised trials | serious ^a^ | serious ^b^ | not serious | serious ^e^ | none | 446/1008 (44.2%) | 445/975 (45.6%) | **RR 0.94** (0.79 to 1.12) | **27 fewer per 1.000** (from 96 fewer to 55 more) | ⨁◯◯◯ VERY LOW | CRITICAL |
| **BPD - birthweight - BW 1000-1250 gram** | | | | | | | | | | | | |
| 8 | randomised trials | serious ^f^ | not serious | not serious | serious ^e^ | none | 159/424 (37.5%) | 152/433 (35.1%) | **RR 1.07** (0.90 to 1.26) | **25 more per 1.000** (from 35 fewer to 91 more) | ⨁⨁◯◯ LOW | CRITICAL |
| **BPD - birthweight - BW 1251-1500 gram** | | | | | | | | | | | | |
| 3 | randomised trials | serious ^a^ | serious ^b^ | not serious | serious ^g^ | none | 9/56 (16.1%) | 12/58 (20.7%) | **RR 0.67** (0.10 to 4.53) | **68 fewer per 1.000** (from 186 fewer to 730 more) | ⨁◯◯◯ VERY LOW | CRITICAL |
| **BPD - birthweight - BW >1500** | | | | | | | | | | | | |
| 1 | randomised trials | serious ^h^ | not serious | not serious | serious ^g^ | none | 1/13 (7.7%) | 2/14 (14.3%) | **RR 0.54** (0.06 to 5.26) | **66 fewer per 1.000** (from 134 fewer to 609 more) | ⨁⨁◯◯ LOW | IMPORTANT |
| **BPD- gestational age - GA < 28 weeks** | | | | | | | | | | | | |
| 9 | randomised trials | serious ^i^ | serious ^b^ | not serious | serious ^e^ | none | 417/948 (44.0%) | 411/910 (45.2%) | **RR 0.92** (0.74 to 1.15) | **36 fewer per 1.000** (from 117 fewer to 68 more) | ⨁◯◯◯ VERY LOW | CRITICAL |
| **BPD- gestational age - GA 28-32 weeks** | | | | | | | | | | | | |
| 15 | randomised trials | serious ^f^ | not serious | not serious | serious ^e^ | publication bias strongly suspected ^d^ | 196/542 (36.2%) | 192/558 (34.4%) | **RR 1.06** (0.92 to 1.21) | **21 more per 1.000** (from 28 fewer to 72 more) | ⨁◯◯◯ VERY LOW | CRITICAL |
| **BPD - PNA - PNA <24** | | | | | | | | | | | | |
| 18 | randomised trials | serious ^a^ | serious ^b^ | not serious | serious ^e^ | publication bias strongly suspected ^d^ | 518/1263 (41.0%) | 511/1247 (41.0%) | **RR 0.94** (0.79 to 1.12) | **25 fewer per 1.000** (from 86 fewer to 49 more) | ⨁◯◯◯ VERY LOW | CRITICAL |
| **BPD - PNA - PNA 24-72h** | | | | | | | | | | | | |
| 4 | randomised trials | serious ^a^ | serious ^b^ | not serious | serious ^g^ | none | 28/96 (29.2%) | 30/94 (31.9%) | **RR 0.85** (0.36 to 2.01) | **48 fewer per 1.000** (from 204 fewer to 322 more) | ⨁◯◯◯ VERY LOW | CRITICAL |
| **BPD - PNA - PNA >72h** | | | | | | | | | | | | |
| 4 | randomised trials | serious ^h^ | not serious | not serious | serious ^g^ | none | 78/152 (51.3%) | 82/153 (53.6%) | **RR 1.01** (0.86 to 1.19) | **5 more per 1.000** (from 75 fewer to 102 more) | ⨁⨁◯◯ LOW | CRITICAL |
| **BPD- crossover - crossover <25%** | | | | | | | | | | | | |
| 3 | randomised trials | serious ^j^ | not serious | not serious | serious ^g^ | none | 113/243 (46.5%) | 107/250 (42.8%) | **RR 1.09** (0.90 to 1.31) | **39 more per 1.000** (from 43 fewer to 133 more) | ⨁⨁◯◯ LOW | CRITICAL |
| **BPD- crossover - crossover 25-50%** | | | | | | | | | | | | |
| 14 | randomised trials | serious ^i^ | serious ^b^ | serious ^c^ | serious ^e^ | publication bias strongly suspected ^d^ | 459/1095 (41.9%) | 456/1065 (42.8%) | **RR 0.94** (0.78 to 1.13) | **26 fewer per 1.000** (from 94 fewer to 56 more) | ⨁◯◯◯ VERY LOW | IMPORTANT |
| **BPD- crossover - crossover >50%** | | | | | | | | | | | | |
| 8 | randomised trials | serious ^a^ | not serious | serious ^c^ | serious ^g^ | none | 51/160 (31.9%) | 58/165 (35.2%) | **RR 0.98** (0.76 to 1.26) | **7 fewer per 1.000** (from 84 fewer to 91 more) | ⨁◯◯◯ VERY LOW | IMPORTANT |
| **BPD - risk of bias - Low risk** | | | | | | | | | | | | |
| 13 | randomised trials | not serious | serious ^b^ | serious ^c^ | serious ^e^ | none | 505/1150 (43.9%) | 498/1134 (43.9%) | **RR 0.97** (0.83 to 1.14) | **13 fewer per 1.000** (from 75 fewer to 61 more) | ⨁◯◯◯ VERY LOW | CRITICAL |
| **BPD - risk of bias - Intermediate risk** | | | | | | | | | | | | |
| 8 | randomised trials | serious ^a^ | not serious | serious ^c^ | serious ^g^ | none | 59/204 (28.9%) | 56/204 (27.5%) | **RR 1.03** (0.74 to 1.44) | **8 more per 1.000** (from 71 fewer to 121 more) | ⨁◯◯◯ VERY LOW | CRITICAL |
| **BPD - risk of bias - High risk** | | | | | | | | | | | | |
| 5 | randomised trials | serious ^a^ | not serious | serious ^c^ | serious ^g^ | publication bias strongly suspected ^d^ | 60/157 (38.2%) | 69/156 (44.2%) | **RR 0.88** (0.68 to 1.13) | **53 fewer per 1.000** (from 142 fewer to 57 more) | ⨁◯◯◯ VERY LOW | IMPORTANT |

**CI:** Confidence interval; **RR:** Risk ratio

#### Explanations

a. Selection bias, no blinding of personnel or incomplete outcome data

b. Heterogeneity >50%

c. Preterm infants with differences in birthweight, gestational age and start of treatment

d. Asymmetrical funnelplot

e. Wide CI

f. Selection bias or no blinding of personnel

g. Number of events <300 and wide CI

h. No blinding of personnel

i. No blinding of personnel or incomplete outcome data

j. Selection bias

Intraventricular hemorrhage grade ≥ 3

**Author(s)**: EJS Jansen, T Hundscheid, W Onland, EMW Kooi, P Andriessen, WP de Boode

**Date**: 2019-09-10

**Question**: Should pharmacological treatment versus placebo/no treatment be used in preterm infants with a PDA to prevent IVH grade ≥ 3?

**Setting**: Preterm infants with a PDA

| **Certainty assessment** | | | | | | | **№ of patients** | | **Effect** | | **Certainty** | **Importance** |
| --- | --- | --- | --- | --- | --- | --- | --- | --- | --- | --- | --- | --- |
| **№ of studies** | **Study design** | **Risk of bias** | **Inconsistency** | **Indirectness** | **Imprecision** | **Other considerations** | **pharamcological treatment** | **placebo/no treatment** | **Relative (95% CI)** | **Absolute (95% CI)** |  |  |
| **Intraventricular hemorrhage (grade ≥ 3)** | | | | | | | | | | | | |
| 19 | randomised trials | serious ^a^ | not serious | serious ^b^ | serious ^c^ | publication bias strongly suspected ^d^ | 157/1532 (10.2%) | 204/1539 (13.3%) | **RR 0.77** (0.64 to 0.94) | **30 fewer per 1.000** (from 48 fewer to 8 fewer) | ⨁◯◯◯ VERY LOW | CRITICAL |
| **IVH grade ≥ 3 - birthweight - BW <1000 gram** | | | | | | | | | | | | |
| 12 | randomised trials | serious ^a^ | not serious | not serious | serious ^e^ | none | 113/1077 (10.5%) | 146/1074 (13.6%) | **RR 0.77** (0.61 to 0.97) | **31 fewer per 1.000** (from 53 fewer to 4 fewer) | ⨁⨁◯◯ LOW | CRITICAL |
| **IVH grade ≥ 3 - birthweight - BW 1000-1250 gram** | | | | | | | | | | | | |
| 6 | randomised trials | serious ^f^ | not serious | not serious | serious ^e^ | none | 42/421 (10.0%) | 55/430 (12.8%) | **RR 0.78** (0.54 to 1.13) | **28 fewer per 1.000** (from 59 fewer to 17 more) | ⨁⨁◯◯ LOW | CRITICAL |
| **IVH grade ≥ 3- gestational age - GA < 28 weeks** | | | | | | | | | | | | |
| 9 | randomised trials | serious ^g^ | not serious | not serious | serious ^e^ | none | 107/1008 (10.6%) | 137/1000 (13.7%) | **RR 0.77** (0.61 to 0.98) | **32 fewer per 1.000** (from 53 fewer to 3 fewer) | ⨁⨁◯◯ LOW | CRITICAL |
| **IVH grade ≥ 3- gestational age - GA 28-32 weeks** | | | | | | | | | | | | |
| 10 | randomised trials | serious ^a^ | not serious | not serious | serious ^e^ | publication bias strongly suspected ^d^ | 50/524 (9.5%) | 67/539 (12.4%) | **RR 0.77** (0.55 to 1.09) | **29 fewer per 1.000** (from 56 fewer to 11 more) | ⨁◯◯◯ VERY LOW | CRITICAL |
| **IVH grade ≥ 3 - PNA - PNA <24** | | | | | | | | | | | | |
| 17 | randomised trials | serious ^a^ | not serious | not serious | serious ^c^ | publication bias strongly suspected ^d^ | 132/1374 (9.6%) | 186/1390 (13.4%) | **RR 0.72** (0.58 to 0.89) | **37 fewer per 1.000** (from 56 fewer to 15 fewer) | ⨁◯◯◯ VERY LOW | CRITICAL |
| **IVH grade ≥ 3 - PNA - PNA 24-72h** | | | | | | | | | | | | |
| 1 | randomised trials | not serious | not serious | not serious | serious ^e^ | none | 6/54 (11.1%) | 7/51 (13.7%) | **RR 0.81** (0.29 to 2.25) | **26 fewer per 1.000** (from 97 fewer to 172 more) | ⨁⨁⨁◯ MODERATE | CRITICAL |
| **IVH grade ≥ 3 - PNA - PNA >72h** | | | | | | | | | | | | |
| 1 | randomised trials | serious ^h^ | not serious | not serious | serious ^e^ | none | 19/104 (18.3%) | 11/98 (11.2%) | **RR 1.63** (0.82 to 3.24) | **71 more per 1.000** (from 20 fewer to 251 more) | ⨁⨁◯◯ LOW | CRITICAL |
| **IVH grade ≥ 3 - open label treatment - open label treatment <25%** | | | | | | | | | | | | |
| 4 | randomised trials | not serious | not serious | serious ^i^ | serious ^e^ | publication bias strongly suspected ^d^ | 26/311 (8.4%) | 26/324 (8.0%) | **RR 1.06** (0.63 to 1.78) | **5 more per 1.000** (from 30 fewer to 63 more) | ⨁◯◯◯ VERY LOW | CRITICAL |
| **IVH grade ≥ 3 - open label treatment - open label treatment 25-50%** | | | | | | | | | | | | |
| 9 | randomised trials | serious ^g^ | not serious | serious ^b^ | serious ^e^ | none | 122/1077 (11.3%) | 159/1075 (14.8%) | **RR 0.76** (0.61 to 0.95) | **35 fewer per 1.000** (from 58 fewer to 7 fewer) | ⨁◯◯◯ VERY LOW | IMPORTANT |
| **IVH grade ≥ 3 - open label treatment - open label treatment >50%** | | | | | | | | | | | | |
| 4 | randomised trials | serious ^f^ | not serious | serious ^b^ | serious ^e^ | none | 8/101 (7.9%) | 14/99 (14.1%) | **RR 0.57** (0.25 to 1.30) | **61 fewer per 1.000** (from 106 fewer to 42 more) | ⨁◯◯◯ VERY LOW | IMPORTANT |
| **IVH grade ≥ 3 - risk of bias - Low risk** | | | | | | | | | | | | |
| 9 | randomised trials | not serious | not serious | serious ^b^ | serious ^e^ | none | 120/1193 (10.1%) | 153/1200 (12.8%) | **RR 0.79** (0.63 to 0.98) | **27 fewer per 1.000** (from 47 fewer to 3 fewer) | ⨁⨁◯◯ LOW | CRITICAL |
| **IVH grade ≥ 3 - risk of bias - Intermediate risk** | | | | | | | | | | | | |
| 7 | randomised trials | serious ^a^ | not serious | serious ^b^ | serious ^e^ | publication bias strongly suspected ^d^ | 17/202 (8.4%) | 32/208 (15.4%) | **RR 0.55** (0.32 to 0.96) | **69 fewer per 1.000** (from 105 fewer to 6 fewer) | ⨁◯◯◯ VERY LOW | IMPORTANT |
| **IVH grade ≥ 3 - risk of bias - High risk** | | | | | | | | | | | | |
| 3 | randomised trials | serious ^a^ | serious ^j^ | serious ^b^ | serious ^e^ | publication bias strongly suspected ^d^ | 20/137 (14.6%) | 19/131 (14.5%) | **RR 1.02** (0.57 to 1.82) | **3 more per 1.000** (from 62 fewer to 119 more) | ⨁◯◯◯ VERY LOW | IMPORTANT |

**CI:** Confidence interval; **RR:** Risk ratio

#### Explanations

a. Selection bias, no blinding of personnel, or incomplete outcome data

b. Preterm infants with differences in birthweight, gestational age, and start of treatment

c. Wide CI

d. Asymmetrical funnel plot

e. Number of events < 300 and wide CI

f. Selection bias and no blinding of personnel

g. No blinding of personnel or incomplete outcome data

h. No blinding of personnel

i. Preterm infants with differences in birthweight

j. Heterogeneity > 50%

Mortality

**Author(s)**: EJS Jansen, T Hundscheid, W Onland, EMW Kooi, P Andriessen, WP de Boode

**Date**: 2019-09-10

**Question**: Should pharmacological treatment versus placebo/no treatment be used in preterm infants with a PDA to prevent mortliaty?

**Setting**: Preterm infants with a PDA

| **Certainty assessment** | | | | | | | **№ of patients** | | **Effect** | | **Certainty** | **Importance** |
| --- | --- | --- | --- | --- | --- | --- | --- | --- | --- | --- | --- | --- |
| **№ of studies** | **Study design** | **Risk of bias** | **Inconsistency** | **Indirectness** | **Imprecision** | **Other considerations** | **pharmacological treatment** | **placebo/no treatment** | **Relative (95% CI)** | **Absolute (95% CI)** |  |  |
| **Mortality** | | | | | | | | | | | | |
| 37 | randomised trials | serious ^a^ | not serious | serious ^b^ | serious ^c^ | none | 319/1978 (16.1%) | 318/2009 (15.8%) | **RR 1.02** (0.89 to 1.17) | **3 more per 1.000** (from 17 fewer to 27 more) | ⨁◯◯◯ VERY LOW | CRITICAL |
| **Mortality - birthweight - BW <1000 gram** | | | | | | | | | | | | |
| 15 | randomised trials | serious ^a^ | not serious | not serious | serious ^c^ | none | 214/1182 (18.1%) | 199/1186 (16.8%) | **RR 1.08** (0.91 to 1.28) | **13 more per 1.000** (from 15 fewer to 47 more) | ⨁⨁◯◯ LOW | CRITICAL |
| **Mortality - birthweight - BW 1000-1250 gram** | | | | | | | | | | | | |
| 15 | randomised trials | serious ^d^ | not serious | not serious | serious ^e^ | none | 88/697 (12.6%) | 92/716 (12.8%) | **RR 0.98** (0.75 to 1.28) | **3 fewer per 1.000** (from 32 fewer to 36 more) | ⨁⨁◯◯ LOW | CRITICAL |
| **Mortality - birthweight - BW 1251-1500 gram** | | | | | | | | | | | | |
| 5 | randomised trials | serious ^a^ | not serious | not serious | serious ^e^ | publication bias strongly suspected ^f^ | 11/76 (14.5%) | 18/79 (22.8%) | **RR 0.65** (0.33 to 1.28) | **80 fewer per 1.000** (from 153 fewer to 64 more) | ⨁◯◯◯ VERY LOW | CRITICAL |
| **Mortality - birthweight - BW >1500** | | | | | | | | | | | | |
| 1 | randomised trials | serious ^g^ | not serious | not serious | serious ^e^ | none | 1/13 (7.7%) | 4/14 (28.6%) | **RR 0.27** (0.03 to 2.11) | **209 fewer per 1.000** (from 277 fewer to 317 more) | ⨁⨁◯◯ LOW | IMPORTANT |
| **Mortality- gestational age - GA < 28 weeks** | | | | | | | | | | | | |
| 12 | randomised trials | serious ^h^ | not serious | not serious | serious ^c^ | publication bias strongly suspected ^f^ | 209/1185 (17.6%) | 198/1184 (16.7%) | **RR 1.05** (0.88 to 1.25) | **8 more per 1.000** (from 20 fewer to 42 more) | ⨁◯◯◯ VERY LOW | CRITICAL |
| **Mortality- gestational age - GA 28-32 weeks** | | | | | | | | | | | | |
| 23 | randomised trials | serious ^a^ | not serious | not serious | serious ^e^ | none | 104/772 (13.5%) | 111/798 (13.9%) | **RR 0.97** (0.76 to 1.24) | **4 fewer per 1.000** (from 33 fewer to 33 more) | ⨁⨁◯◯ LOW | CRITICAL |
| **Mortality- PNA - PNA <24** | | | | | | | | | | | | |
| 24 | randomised trials | serious ^a^ | not serious | not serious | serious ^c^ | none | 269/1584 (17.0%) | 271/1613 (16.8%) | **RR 1.01** (0.87 to 1.17) | **2 more per 1.000** (from 22 fewer to 29 more) | ⨁⨁◯◯ LOW | CRITICAL |
| **Mortality- PNA - PNA 24-72h** | | | | | | | | | | | | |
| 4 | randomised trials | serious ^a^ | not serious | not serious | serious ^e^ | none | 8/76 (10.5%) | 11/77 (14.3%) | **RR 0.75** (0.32 to 1.77) | **36 fewer per 1.000** (from 97 fewer to 110 more) | ⨁⨁◯◯ LOW | CRITICAL |
| **Mortality- PNA - PNA >72h** | | | | | | | | | | | | |
| 9 | randomised trials | not serious | not serious | not serious | serious ^e^ | none | 42/318 (13.2%) | 36/319 (11.3%) | **RR 1.21** (0.81 to 1.81) | **24 more per 1.000** (from 21 fewer to 91 more) | ⨁⨁⨁◯ MODERATE | CRITICAL |
| **Mortality- crossover - crossover <25%** | | | | | | | | | | | | |
| 6 | randomised trials | serious ^i^ | not serious | serious ^b^ | serious ^e^ | publication bias strongly suspected ^f^ | 56/377 (14.9%) | 51/392 (13.0%) | **RR 1.14** (0.81 to 1.62) | **18 more per 1.000** (from 25 fewer to 81 more) | ⨁◯◯◯ VERY LOW | CRITICAL |
| **Mortality- crossover - crossover 25-50%** | | | | | | | | | | | | |
| 14 | randomised trials | serious ^h^ | not serious | serious ^b^ | serious ^c^ | none | 229/1223 (18.7%) | 220/1234 (17.8%) | **RR 1.05** (0.89 to 1.24) | **9 more per 1.000** (from 20 fewer to 43 more) | ⨁◯◯◯ VERY LOW | IMPORTANT |
| **Mortality- crossover - crossover >50%** | | | | | | | | | | | | |
| 12 | randomised trials | serious ^a^ | not serious | serious ^b^ | serious ^e^ | none | 30/247 (12.1%) | 36/252 (14.3%) | **RR 0.86** (0.55 to 1.33) | **20 fewer per 1.000** (from 64 fewer to 47 more) | ⨁◯◯◯ VERY LOW | IMPORTANT |
| **Mortality - risk of bias - Low risk** | | | | | | | | | | | | |
| 19 | randomised trials | not serious | not serious | serious ^b^ | serious ^c^ | none | 244/1458 (16.7%) | 241/1482 (16.3%) | **RR 1.03** (0.88 to 1.21) | **5 more per 1.000** (from 20 fewer to 34 more) | ⨁⨁◯◯ LOW | CRITICAL |
| **Mortality - risk of bias - Intermediate risk** | | | | | | | | | | | | |
| 12 | randomised trials | serious ^a^ | not serious | serious ^b^ | serious ^e^ | publication bias strongly suspected ^f^ | 50/343 (14.6%) | 53/351 (15.1%) | **RR 0.98** (0.70 to 1.38) | **3 fewer per 1.000** (from 45 fewer to 57 more) | ⨁◯◯◯ VERY LOW | CRITICAL |
| **Mortality - risk of bias - High risk** | | | | | | | | | | | | |
| 6 | randomised trials | serious ^a^ | not serious | serious ^b^ | serious ^e^ | publication bias strongly suspected ^f^ | 25/177 (14.1%) | 24/176 (13.6%) | **RR 1.05** (0.62 to 1.77) | **7 more per 1.000** (from 52 fewer to 105 more) | ⨁◯◯◯ VERY LOW | IMPORTANT |

**CI:** Confidence interval; **RR:** Risk ratio

#### Explanations

a. Selection bias, no blinding of personnel or incomplete outcome data

b. Preterm infants with differences in birthweight, gestational age and start of treatment

c. Wide CI

d. Selection bias or no blinding of personnel

e. Number of events <300 and wide CI

f. Asymmetrical funnelplot

g. No blinding of personnel

h. No blinding or incomplete outcome data

i. Selection bias or incomplete outcome data

**Supplement 3**

PRISMA 2009 CHECKLIST

| **Section/topic** | **#** | **Checklist item** | **Reported on page #** |
| --- | --- | --- | --- |
| **TITLE** | | |  |
| Title | 1 | Identify the report as a systematic review, meta-analysis, or both. | 1 |
| **ABSTRACT** | | |  |
| Structured summary | 2 | Provide a structured summary including, as applicable: background; objectives; data sources; study eligibility criteria, participants, and interventions; study appraisal and synthesis methods; results; limitations; conclusions and implications of key findings; systematic review registration number. | 2 |
| **INTRODUCTION** | | |  |
| Rationale | 3 | Describe the rationale for the review in the context of what is already known. | 3 |
| Objectives | 4 | Provide an explicit statement of questions being addressed with reference to participants, interventions, comparisons, outcomes, and study design (PICOS). | 3 |
| **METHODS** | | |  |
| Protocol and registration | 5 | Indicate if a review protocol exists, if and where it can be accessed (e.g., Web address), and, if available, provide registration information including registration number. | - |
| Eligibility criteria | 6 | Specify study characteristics (e.g., PICOS, length of follow-up) and report characteristics (e.g., years considered, language, publication status) used as criteria for eligibility, giving rationale. | 3 |
| Information sources | 7 | Describe all information sources (e.g., databases with dates of coverage, contact with study authors to identify additional studies) in the search and date last searched. | 3 |
| Search | 8 | Present full electronic search strategy for at least one database, including any limits used, such that it could be repeated. | 3+suppl 1 |
| Study selection | 9 | State the process for selecting studies (i.e., screening, eligibility, included in systematic review, and, if applicable, included in the meta-analysis). | 3 |
| Data collection process | 10 | Describe method of data extraction from reports (e.g., piloted forms, independently, in duplicate) and any processes for obtaining and confirming data from investigators. | 4 |
| Data items | 11 | List and define all variables for which data were sought (e.g., PICOS, funding sources) and any assumptions and simplifications made. | 4 |
| Risk of bias in individual studies | 12 | Describe methods used for assessing risk of bias of individual studies (including specification of whether this was done at the study or outcome level), and how this information is to be used in any data synthesis. | 4 |
| Summary measures | 13 | State the principal summary measures (e.g., risk ratio, difference in means). | 4 |
| Synthesis of results | 14 | Describe the methods of handling data and combining results of studies, if done, including measures of consistency (e.g., I^2^) for each meta-analysis. | 4 |
| Risk of bias across studies | 15 | Specify any assessment of risk of bias that may affect the cumulative evidence (e.g., publication bias, selective reporting within studies). | 4-5 |
| Additional analyses | 16 | Describe methods of additional analyses (e.g., sensitivity or subgroup analyses, meta-regression), if done, indicating which were pre-specified. | 4 |
| **RESULTS** | | |  |
| Study selection | 17 | Give numbers of studies screened, assessed for eligibility, and included in the review, with reasons for exclusions at each stage, ideally with a flow diagram. | 5 |
| Study characteristics | 18 | For each study, present characteristics for which data were extracted (e.g., study size, PICOS, follow-up period) and provide the citations. | 5+ table 1 |
| Risk of bias within studies | 19 | Present data on risk of bias of each study and, if available, any outcome level assessment (see item 12). | 5 + table 1 |
| Results of individual studies | 20 | For all outcomes considered (benefits or harms), present, for each study: (a) simple summary data for each intervention group (b) effect estimates and confidence intervals, ideally with a forest plot. | 5-6 |
| Synthesis of results | 21 | Present results of each meta-analysis done, including confidence intervals and measures of consistency. | 5-6 + table 2 |
| Risk of bias across studies | 22 | Present results of any assessment of risk of bias across studies (see Item 15). | 5-6 |
| Additional analysis | 23 | Give results of additional analyses, if done (e.g., sensitivity or subgroup analyses, meta-regression [see Item 16]). | 5-6 |
| **DISCUSSION** | | |  |
| Summary of evidence | 24 | Summarize the main findings including the strength of evidence for each main outcome; consider their relevance to key groups (e.g., healthcare providers, users, and policy makers). | 6 |
| Limitations | 25 | Discuss limitations at study and outcome level (e.g., risk of bias), and at review-level (e.g., incomplete retrieval of identified research, reporting bias). | 8 |
| Conclusions | 26 | Provide a general interpretation of the results in the context of other evidence, and implications for future research. | 8 |
| **FUNDING** | | |  |
| Funding | 27 | Describe sources of funding for the systematic review and other support (e.g., supply of data); role of funders for the systematic review. | 9 |
